# Decipher the Mechanisms of Protein Conformational Changes Induced by Nucleotide Binding through Free-Energy Landscape Analysis: ATP Binding to Hsp70

**DOI:** 10.1371/journal.pcbi.1003379

**Published:** 2013-12-12

**Authors:** Adrien Nicolaï, Patrice Delarue, Patrick Senet

**Affiliations:** Laboratoire Interdisciplinaire Carnot de Bourgogne, UMR 6303 CNRS-Université de Bourgogne, Dijon, France; UNC Charlotte, United States of America

## Abstract

ATP regulates the function of many proteins in the cell by transducing its binding and hydrolysis energies into protein conformational changes by mechanisms which are challenging to identify at the atomic scale. Based on molecular dynamics (MD) simulations, a method is proposed to analyze the structural changes induced by ATP binding to a protein by computing the effective free-energy landscape (FEL) of a subset of its coordinates along its amino-acid sequence. The method is applied to characterize the mechanism by which the binding of ATP to the nucleotide-binding domain (NBD) of Hsp70 propagates a signal to its substrate-binding domain (SBD). Unbiased MD simulations were performed for Hsp70-DnaK chaperone in nucleotide-free, ADP-bound and ATP-bound states. The simulations revealed that the SBD does not interact with the NBD for DnaK in its nucleotide-free and ADP-bound states whereas the docking of the SBD was found in the ATP-bound state. The docked state induced by ATP binding found in MD is an intermediate state between the initial nucleotide-free and final ATP-bound states of Hsp70. The analysis of the FEL projected along the amino-acid sequence permitted to identify a subset of 27 protein internal coordinates corresponding to a network of 91 key residues involved in the conformational change induced by ATP binding. Among the 91 residues, 26 are identified for the first time, whereas the others were shown relevant for the allosteric communication of Hsp70 s in several experiments and bioinformatics analysis. The FEL analysis revealed also the origin of the ATP-induced structural modifications of the SBD recently measured by Electron Paramagnetic Resonance. The pathway between the nucleotide-free and the intermediate state of DnaK was extracted by applying principal component analysis to the subset of internal coordinates describing the transition. The methodology proposed is general and could be applied to analyze allosteric communication in other proteins.

## Author Summary

The precise biophysical characterization of the mechanisms of the protein conformational changes controlled by a nucleotide remains a challenge in biology. Molecular dynamics simulations of proteins in different nucleotide-binding states contain information on the nucleotide-dependent conformational dynamics. However, it is difficult to extract relevant information about the conformation-induced mechanism from the raw molecular dynamics data. Herein, we addressed this issue for the major ATP-dependent molecular chaperones Hsp70 s, which contribute to crucial cellular processes and are involved in several neurodegenerative diseases and in cancer. To function, Hsp70 undergoes several conformational changes controlled by the state of its nucleotide-binding domain. We demonstrated that the analysis of the effective free-energy landscape of the protein projected along the amino-acid sequence and computed from the molecular dynamics simulations of Hsp70 in different nucleotide-binding states, holds the key to identify the key residues of the conformational induced pathway. Identification of the key residues involved in the propagation of the structural changes induced by ATP binding offer alternative druggable specific sites other than the ligand binding clefts. The methodology developed for Hsp70 is general and can be adapted to any ligand induced conformational change in proteins.

## Introduction

Biomolecular machines, as motor proteins [1] and ATP-dependent molecular chaperones [2], undergo a major conformational change upon ATP binding and hydrolysis. In many cases, the ATP molecule regulates the protein function by an allosteric mechanism [3]–[7]: binding of ATP in the ATPase domain of these proteins transmits a conformational change to a distant binding site where the substrate binding propagates an analogous conformational change towards the nucleotide-binding site. The exact biophysical characterization of the mechanisms of propagation of a conformational change induced by ligand binding through a protein remains a major challenge in structural biology [8], [9]. In biomolecular machines, as the ATP-dependent molecular chaperones [10], crystallographic data of the different nucleotide-bound states do not provide generally a complete description of the mechanism by which the protein undergoes a conformational change, because the pathway between the different conformational states and the dynamical information are missing [5].

In recent years, NMR, among other techniques, contributed to the characterization of allostery and protein dynamics [8], [9], [11] including nucleotide-dependent conformational changes in molecular chaperones [12], [13]. Conformational changes induced by a ligand can be mediated by perturbations of the mean protein conformation (enthalpic effect) as well as by changes of its fluctuations and dynamics (entropic effect) [5], [9], [14]–[16]. Both enthalpic and entropic effects are encompassed in the free-energy landscape (FEL) of a protein [17]. In solution, the FEL is best regarded as a multi-dimensional surface with multiple local minima separated by barriers [17]. Different protein-bound states correspond to different FELs, with different shapes and distributions of populations of conformers [4], [5]. Hence, the nucleotide binding and the nucleotide release in a biomolecular machine redistribute its conformational substates and modify its FEL [18].

In many cases, how the dynamics contributes to the conformational transition at the different time-scales of the protein motions and how the FEL is modified upon nucleotide binding remain elusive. Nowadays, standard all-atom molecular dynamics (MD) simulations [19]–[21] represent a powerful tool, complementary to experiments, for investigating the conformational sampling and nucleotide-dependent conformations of biomolecular machines [22]–[25]. The fully unbiased all-atom MD simulations permit to monitor the sequence of *spontaneous* conformational changes at the atomic level [26] and to translate the conformational fluctuations in terms of modified protein FEL. Although unbiased MD simulations of a fully solvated protein may extend to the millisecond range [27] with specific hardware [28], the current state-of-art of unbiased MD simulations correspond typically to 100 ns up to 1 µs time-scale for a system of several thousands of atoms in explicit solvent with the standard computational capabilities available to most of the research groups [29]–[31]. However, transition between active and inactive states in proteins occurs typically on the microsecond to the millisecond time-scales [32]. The complete conformational transition between different nucleotide-binding states of a molecular chaperone in fully unconstrained all-atom MD is thus still inaccessible [31]. To overcome this time-scale restriction, different computational strategies were developed to shorten artificially the time-scale of the conformational transitions. Targeted MD [33], accelerated MD [34], metadynamics [35] to cite a few allow the acceleration of the conformational sampling of all-atom MD by adding a bias potential. An alternative strategy consists to eliminate the fast degrees of freedom of the protein from the MD calculation by coarse-graining the protein structure. A recent application to the 70 kDa heat-shock protein (Hsp70), a key ubiquitous molecular chaperone [36], allow us to observe a short-life time full transition between an ATP-like state and an ADP-like state using a coarse-grained model where the solvent and the nucleotides were implicit [37]. However, current coarse-grained models of proteins lack the description of the crucial explicit interactions between the nucleotide and the protein which can be only described accurately at the atomic level.

In spite the fact that all-atom unbiased MD simulations are not able to follow the full conformational change of a large ATP-dependent protein, as Hsp70 analyzed in the present work, MD simulations probing the structural fluctuations around the end points of a conformational transition are useful to understand the conformational changes. Normal mode analysis (NMA) has been also extensively applied to study the conformational transitions in proteins because the directions of the low-frequency vibrational modes provide the direction of the largest deformation at thermal equilibrium and can serve as collective coordinates to describe the conformational changes [38], [39]. Indeed, intrinsic low-frequency vibrational dynamics of proteins in different conformational states correlates with the structural changes between the different conformations induced by ligand binding or by protein-protein interactions [16], [40]–[44].

Aside the limited sampling issue of standard all-atom MD simulations of large conformational changes upon ligand binding, another issue is to extract from these data relevant information about the mechanism of propagation of a small perturbation at one ligand binding site to a different remote binding site. To get insight into the mechanisms, several analytical tools were applied to ATP-dependent conformational transitions such as the analysis of contact maps [45] and of the correlations between the residue motions [22]. In NMA, relevant information is gained by projecting the direction of the low-frequency modes of a given conformation on the shortest path between the two end structures of a conformational transition [1], [23], [46]–[49]. None of these approaches is easily related simultaneously on the FEL of the protein and to mutagenesis experimental data.

In the present work, we addressed these two issues (MD sampling and relation between FEL and biochemical data) for the conformational change induced upon nucleotide binding to a molecular chaperone Hsp70 by using rather extensive unbiased MD simulations of the protein in three different nucleotide-binding states; nucleotide-free, ADP-bound and ATP-bound. Hsp70 is a key molecular chaperone which assists in the correct folding and refolding of proteins in all prokaryotic and eukaryotic organisms [2], [36], [50]–[53]. Hsp70 s contribute to other crucial cellular processes including the protein degradation, the translocation of peptides across the cell membrane, the formation of protein complexes, and the inhibition of the programmed cell death (apoptosis) [54]–[57]. Among the Hsp70 s, *human* Hsp70 (hHsp70) has attracted great interest because of its demonstrated implications in numerous misfolding diseases [58] (Parkinson, Alzheimer…) and in cancer [59]. Hsp70 s of all species share common structural features and it is hypothesized that they perform their biological functions in a similar manner. Most mechanistic information about hHsp70 was derived from comparisons with the highly homologous bacterial *E. coli* Hsp70 (DnaK) (see Table S1). It should be mentioned however that a high homology of sequence between two proteins does not guarantee that their allosteric mechanisms and residues involved are similar [60]. In the present work, the calculations and analysis were performed for DnaK because a large set of structural and biochemical data are available (see Results and Discussion sections for a comparison with the present results and their implications for hHsp70). In particular, the allosteric communication in Hsp70 s, i.e. how the binding of a ligand in the nucleotide-binding domain (NBD) modifies the conformation of the remote substrate-binding domain (SBD) of the protein, was extensively studied for DnaK [12], [61]–[69]. The structural model used in the present work is the two-domain structure of ADP-bound DnaK (residues 4–603, missing the C-terminal part) solved by NMR (PDB ID: 2KHO) [70]. Our first aim was to probe the conformational dynamics and the structural perturbations induced upon exchange of ADP by ATP and by the removal of ADP from this NMR model of *E. coli* DnaK on the micro-second time-scale at the atomic scale.

The sampling issue of the unbiased MD simulations of *E. coli* DnaK was addressed by considering two trajectories with different initial conditions of the protein in each of its nucleotide-binding states and more important by restricting the data to a *subset* of the conformational degrees of freedom for which the convergence between the two trajectories was reached. This was achieved by projecting the FEL of the protein along its amino-acid sequence by computing the free-energy profiles (FEPs) along the coarse-grained dihedral angles (CGDAs) *γ* defined from four consecutive C^α^ atoms of the protein (Fig. S1) and describing the backbone motions [71], [72]. Only the FEPs which were similar in the two trajectories of the protein in the same nucleotide-binding state were kept for the analysis of the allosteric communication, we named these FEPs the DATA. No information can be exploited from the other FEPs: they may or not be relevant to the conformational fluctuations of the protein (NO DATA set). With this approach, we were able to quantify how each FEP varies along the amino-acid sequence depending on the nucleotide-binding state of the protein and to relate the FEP modifications to biochemical data of wild-type or mutant *E. coli* DnaK chaperones. Indeed, the comparison between the DATA sets of FEPs for *E. coli* DnaK in different nucleotide-binding states lead finally to a subset of only 27 key dihedral angles involved in the conformational nucleotide-dependent transitions. The fluctuations of these 27 CGDAs *γ* on their FEPs are subdiffusive [71]–[73]. The coupling between the CGDAs *γ* can be revealed by analyzing the correlation of their motions using the dihedral principal component analysis (dPCA) [74] allowing the definition of collective motions to which these degrees of freedom contribute the most, in a similar manner to principal component analysis in Cartesian coordinates [75], [76] or to the full-correlation analysis based on the information theory [77]. The study of the FELs was complemented by an analysis of the long-life time contacts between residues present in ATP-DnaK trajectories and not present in the trajectories of the protein in the other nucleotide-binding states.

The paper is organized as follows. Results are presented in the next section and are compared to available experimental data. The structure of Hsp70 s and the results of the unbiased all-atom MD simulations for *E. coli* DnaK are shown. There, we described and compared to available experimental data an intermediate state found by MD upon ATP-binding, named ATP*-Hsp70, where the two domains of the protein are docked on a sub-microsecond time-scale. The construction of a map of persistent residue contacts in ATP-bound DnaK conformational dynamics is described and compared to available experimental data. Next, the methodology to cope with the limited sampling of the present unbiased MD simulations and to extract the key residues from the analysis of the FEPs along the amino-acid sequence is presented, as well as its application to the MD simulations of ATP-bound DnaK. Then, a comparison between the key residues extracted from this new methodology with biochemical and structural data on the allostery in Hsp70 s is discussed in detail. The paper ends with a short conclusion where the major results are summarized and with the Methods section.

## Results

### Conformational changes of *E. coli* Hsp70 (DnaK)

#### Conformations of Hsp70 s from experiments

**Figure 1:**
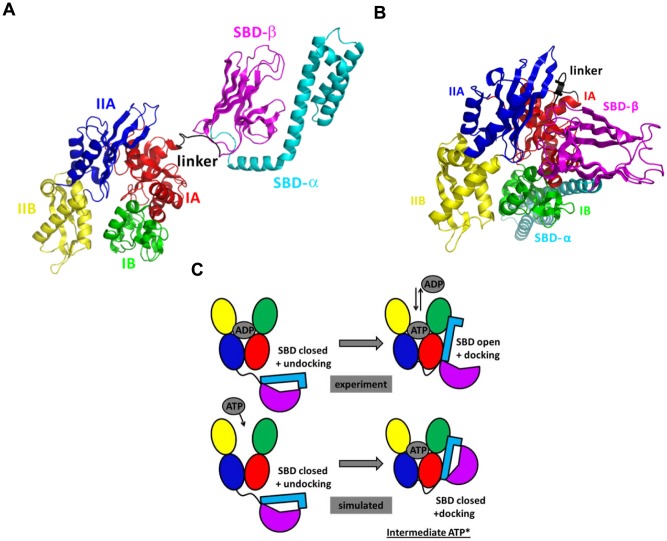
Nucleotide-dependent conformational states of Hsp70 s. A: Cartoon diagram of the experimental NMR structure of *E. coli* ADP-Hsp70 (PDB ID: 2KHO) [70] used as input structure for the MD simulations. B: Cartoon diagram of the experimental X-ray structure of *E. coli* ATP-Hsp70 (PDB ID: 4B9Q) [69]. C: Schematic representation of the conformational transition as expected from experimental data and observed in MD simulations of ATP-Hsp70, starting from the closed conformation of Hsp70. The color code is the following: NBD-IA (residues 4–37, 112–182 and 363–383; red), NBD-IB (residues 38–111; green), NBD-IIA (residues 183–227 and 311–362; blue), NBD-IIB (residues 228–310; yellow), linker (residues 384–393; black), SBD-β (residues 394–502; magenta) and SBD-α (residues 503–603; cyan). These figures were prepared with PyMOL [http://www.pymol.org].

All homologous Hsp70 s share a common architecture composed of a 45 kDa N-terminal NBD [78] which catalyzes ATP hydrolysis, and of a 25 kDa C-terminal domain [79] which is divided into SBD, which binds to non-native or unfolded substrate proteins, and a C-unstructured terminal domain (Fig. 1 A). The N-terminal 45-kDa NBD is divided into two rather symmetrical lobes, I and II (Fig. 1 A), each divided into two subdomains A and B [78]. The SBD is composed of a β-sandwich subdomain (SBD-β), which has a hydrophobic peptide binding pocket, and of a α-helical “lid” subdomain (SBD-α), which regulates the access of the substrate to the SBD-β [79]. The NBD and the SBD are connected by a conserved hydrophobic linker [80].

Hsp70 s occur in two main conformations to perform their chaperone function, we named closed conformation (with ADP bound to the NBD or without nucleotide = APO, Fig. 1 A) and open conformation (with ATP bound to the NBD, Fig. 1 B). In the nucleotide-free Hsp70 and in ADP-bound Hsp70, the SBD is assumed to be closed with low binding and release rate of the protein substrate. However, recent experiments shown that the complete closure of the SBD is not strictly required in the chaperone protein interactions [81]. In absence of ATP, the SBD and the NBD are undocked and the inter-domain linker is exposed to solvent, as shown by low-resolution Small Angle X-ray Scattering (SAXS) data [82], [83], Förster resonance energy transfer (FRET) data [84], [85] and by the two-domain Hsp70 NMR derived-structure (PDB ID: 2KHO) [70] (Fig. 1 A). In ATP-bound Hsp70, the SBD is open with fast binding and release of the protein substrate, and the SBD and NBD are docked, as shown by SAXS data [82], [83] and FRET data [84], [85] and suggested by the X-ray structure of an ATP-Hsp110 homolog [86], [87] and by a new open conformation of an ATP-bound DnaK mutant (PDB ID: 4B9Q) [69] stabilized by introducing disulphide bridges at specific positions deduced from the structure of the ATP-Hsp110 homolog (Fig. 1 B).

The transition between the open and the closed structural states of Hsp70 is triggered by the ATP binding to the NBD which induces a conformational change of the inter-domain linker and the opening of the SBD [61], [62], through an allosteric mechanism which is not fully elucidated [12], [61]–[68]. The ATP hydrolysis restores the closed conformation of the SBD [59], [60]. In *vivo*, the ATP hydrolysis and the nucleotide exchange rates are increased by co-chaperones [88].

#### Unbiased MD simulations of the APO and ADP-bound DnaK reveal that the SBD is moving freely around the NBD whereas MD simulations of the ATP-bound DnaK reveal the docking of the SBD onto the NBD

To get insights into the mechanism of the conformational dynamics of *E. coli* DnaK, we focused here on the spontaneous conformational transition, i.e. from the closed to the open conformation (Fig. 1 C), induced by the binding of ATP to the NBD of DnaK in absence of protein substrate [61], [62]. We performed 2 unbiased all-atom MD simulations in explicit solvent with different initial conditions in each nucleotide-binding state of the NBD, i.e. nucleotide-free (APO state), ADP-bound and ATP-bound for a total simulation time of 1.9 µs. The six MD trajectories (APO1, APO2, ADP1, ADP2, ATP1, and ATP2) are described in the Methods section.

**Figure 2:**
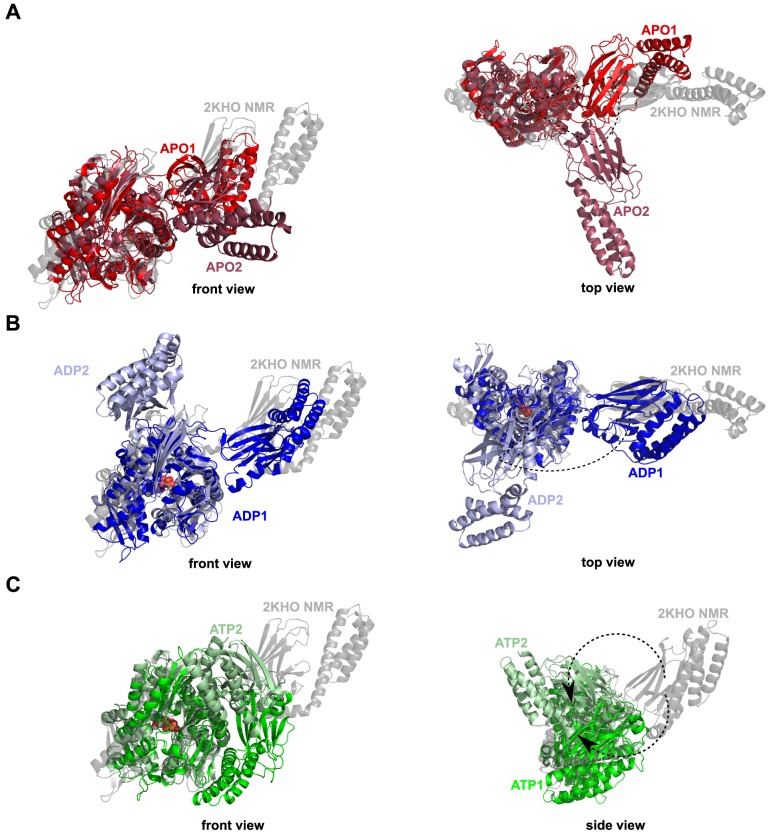
Cartoon representation of the representative structures obtained by MD simulations. A: MD runs APO1 (red) and APO2 (firebrick). B: MD runs ADP1 (blue) and ADP2 (light blue). C: MD runs ATP1 (green) and ATP2 (pale green). The NMR-derived structure used as starting point for the MD simulations (PDB ID: 2KHO) [70] is shown in transparent gray. Representative structures are extracted from a dPCA on the CGDAs *γ* (see Methods section). These figures were prepared with PyMOL [http://www.pymol.org].

First, MD simulations of the nucleotide-free and the ADP-bound DnaK revealed that the SBD is moving freely around the NBD. Indeed, Fig. 2 clearly shows that the different conformational sub-states of APO (Fig. 2 A) and ADP-DnaK (Fig. 2 B) correspond to different orientations of the SBD relative to the NBD. Structures of *E. coli* DnaK upon ADP-binding and without nucleotide are elongated with no strong interactions between the two main domains, as expected by comparison with experimental data and particularly SAXS experiments [83]. In addition, Figs. 2 A and 2 B agree with the hypothesis of a SBD diffusing around a cone, as suggested from residual dipolar coupling NMR data of *E. coli* DnaK [70]. By contrast, MD simulations of ATP-bound DnaK reveal conformational changes. Indeed, significant structural changes and particularly the docking of the SBD onto the NBD (more precisely on the lobe I of the NBD) were found upon the binding of ATP (Fig. 2 C) in the two MD runs ATP1 and ATP2. In addition, differences between the MD runs ATP1 and ATP2 were observed. Actually, it seems that there are (at least) two different binding positions of the SBD onto the lobe I of the NBD, more precisely there are two orientations of the SBD, bound to the NBD (Fig. 2 C). The time-scale of the present MD simulations is too short to observe the full conformational change between the ADP-bound and the ATP-bound states of Hsp70: in other words, the opening of the substrate binding domain was not observed (Fig. 2 C). However, we found that the ATP-binding to the NBD of *E. coli* DnaK induces the docking of a closed SBD onto the NBD (we named this intermediate state ATP*) whereas this docking was not observed for ADP-bound DnaK and for APO-DnaK.

#### Comparison of ATP*-DnaK structure with experimental and MD data

**Figure 3:**
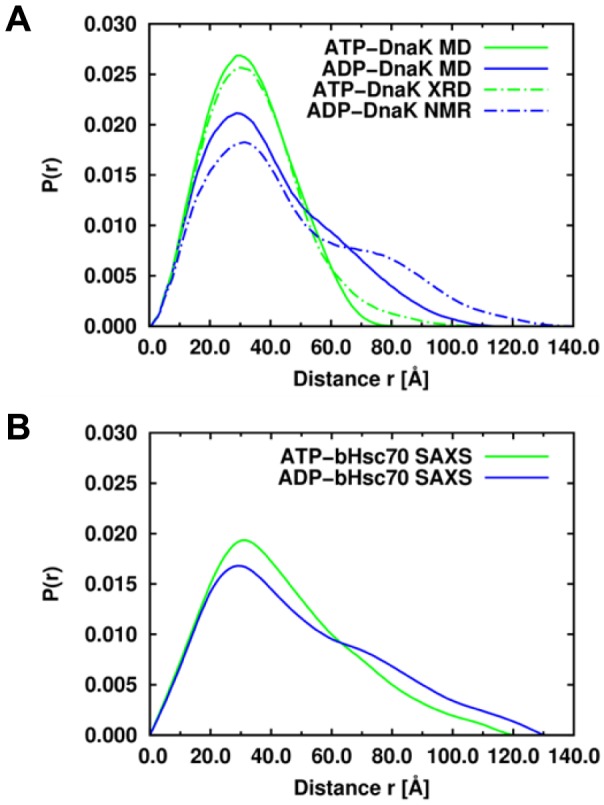
Comparison between the simulated and experimental distance distribution functions *P(r)* of Hsp70 s. A: Distance distribution function *P(r)* of the ATP-bound (green lines) and ADP-bound (blue lines) of *E. coli* DnaK computed from MD simulations (solid lines) and computed from the experimental X-ray (PDB ID: 4BQ9) [69] and NMR-derived (PDB ID: 2KHO) [70] structures (dashed lines), respectively. B: Distance distribution function *P(r)* of the ATP-bound (green line) and ADP-bound (blue line) of bHsc70. The function *P(r)* of bHsc70 in the ATP and ADP states were digitized from Figure 4 of Ref. [82].

In spite of the fact that the SBD of ATP*-DnaK remains closed on the time-scale of the present MD simulations, its structural properties are compatible with SAXS [82], [83], as shown in Fig. 3. In all SAXS experiments [82], [83], [89], the structure of ATP-Hsp70 was more compact than the structure of ADP-Hsp70: the difference between their radius of gyration, *ΔR_g_* = *R_g_(ADP)*−*R_g_(ATP)*, was about 5 Å for *E. coli* DnaK [83] and between 2 and 7 Å for bovine Hsc70 (bHsc70) [82], [89]. In our MD simulations of *E. coli* DnaK, *ΔR_g_* varies between 3.6 Å and 6.8 Å in good agreement with SAXS data. The distribution of the distance *r* between two heavy atoms, *P(r)*, in ATP-bound DnaK and ADP-bound DnaK computed from MD simulations are also in good agreement with the distribution *P(r)* of Hsp70 homologs measured by SAXS and with the distribution *P(r)* of *E. coli* DnaK computed from the experimental X-ray [69] and NMR [70] resolved 3D structures (Fig. 3). In MD, *P(r)* has only one peak at about 30 Å for ATP-DnaK (Fig. 3 A), in agreement with the distribution extracted from the SAXS data of ATP-bHsc70 [82] (Fig. 3 B) and from the X-ray data of ATP-DnaK [69] (Fig. 3 A). On the contrary, the functions *P(r)* extracted from SAXS data of ADP-bHsc70 [82] (Fig. 3 B) and from NMR data of ADP-DnaK [70] (Fig. 3 A) and the one calculated from the MD simulations of ADP-DnaK (Fig. 3 A) show two peaks: one at 30 Å and a distinct shoulder at 60 Å, reflecting the bilobal shape of the protein in this nucleotide-binding state.

The intermediate structure of ATP*-DnaK is different from the very recent crystallized triple mutant ATP-bound DnaK (E47C, T199A, F529C) in an *open* conformational state with a SBD-α covalently bound to the NBD through a disulphide bridge 47C-529C (PDB ID: 4B9Q) [69]. In ATP*, as in ATP-bound DnaK (Fig. 1 B), the linker forms a β strand interacting with the loop between the strands β2 and β2' of the SBD-β. However, the position of the linker in ATP-bound DnaK is shifted relative to the one in ATP*: a contact is formed between D393 and I418 in ATP-bound structure whereas a contact is formed between D393 and N415 in ATP* (see next subsection) [69].

Recent NMR studies suggest that DnaK occur in at least three states: a closed conformation (ADP-bound with a flexible linker as the NMR-derived structure [70]), an open conformation (ATP-bound with the linker bound between the subdomains IA/IIA of the NBD and the NBD/SBD docked as in the crystallized structure [69]), and an intermediate allosteric active state (where the closed SBD *bound to a substrate* and the NBD domains interact with the linker bound to the NBD). This intermediate state is however different from ATP*-DnaK found in MD as it occurred when both ATP and the protein substrate are bound to the NBD and to the SBD, respectively.

The intermediate state ATP*-DnaK was the most frequent SBD/NBD transient docked state found in our recent intensive coarse-grained MD simulations of *E. coli* DnaK using a completely unrelated force-field [37]. On the contrary, in the recent all-atom MD simulations of DnaK by Chiappori et al. [25] using the same force-field used in the present work, the authors did not observe the docking of the SBD onto the NBD. They did find however a motion of the SBD towards the NBD (Fig. 2 in Ref. 25) which resembles to the one leading to the ATP*-DnaK intermediate state found here. It is worth noting however that the number and the duration of the MD simulations in Ref. 25 were much smaller than the present ones: the trajectories were typically of about 50–100 ns of duration with the longest duration being 225 ns for an ATP-bound DnaK trajectory [25]. The absence of ATP* in the calculations of Ref. 25 may arise simply from the too short duration used in this earlier work [25] for the random initial condition chosen.

### Specific contacts in ATP-bound DnaK

In order to characterize the interdomain interactions leading to the ATP*-DnaK intermediate state (Fig. 2 C), as well as the interactions between the nucleotide and the NBD of *E. coli* DnaK, the contact maps (CMs) of ADP and ATP-bound DnaK and the contact map difference (CMD) ATP/ADP were computed for the MD trajectories of *E. coli* DnaK in these two different nucleotide-binding states (see Methods section for the calculations of CM and CMD).

**Figure 4:**
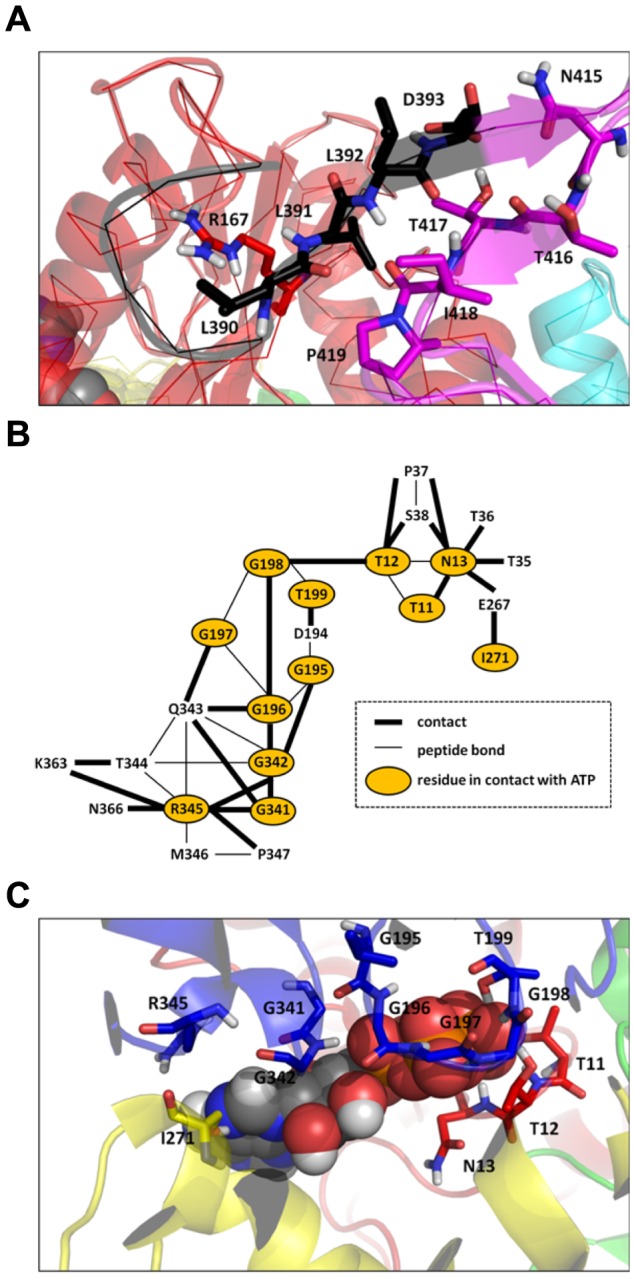
Contact maps from ATP-bound and ADP-bound MD trajectories. A: Interdomain interactions extracted from the contact map difference ATP/ADP (see text for details). Residues involved in the interdomain interactions are shown in stick representation. The color code is the same as in Fig. 1. B: Interactions between the ATP nucleotide and the NBD of DnaK from ATP-bound MD trajectories. C: Three-dimensional representation of the interactions between ATP and the NBD. Residues of the NBD in contact with ATP are shown in stick representation. The color code of the protein structure is the same as in Fig. 1. These figures were prepared with PyMOL [http://www.pymol.org].

The CM of ATP-bound DnaK (Fig. S2 A), which is composed of 1994 contacts, contains only 6 interdomain contacts (contacts between NBD/linker, linker/SBD or NBD/SBD): R167-L390, R167-L391, L391-T417, L391-P419, L392-T416 and D393-N415 (Fig. 4 A). The CM of ADP-bound DnaK has no interdomain contacts (Fig. S2 B), which means that the interdomain contacts are unique to the ATP* intermediate state, as revealed by the CMD ATP/ADP (Fig. S2 C). In MD, this major difference about the interdomain contacts in ADP-bound and in ATP-bound DnaK arises from a different interaction between the NBD and the linker in these two nucleotide-binding states, as shown by the different distances R167-L390 and R167-L391 in these two states (Table 1).

**Table 1:** List of the interdomain interactions and their corresponding average distances extracted from the contact map difference ATP/ADP (see text for details).

Type	Residues	Distance (Å)	
		ATP	ADP
NBD/Linker	R167-L390	3.5	12.5
	R167-L391	3.8	13.4
Linker/SBD	L391-T417	3.7	4.6
	L391-P419	3.7	5.0
	L392-T416	3.6	4.4
	D393-N415	3.2	4.1

The comparison between MD results for ADP-bound and ATP-bound DnaK shows that the communication between the NBD, the linker and the SBD involves the residue R167 of the subdomain IA of the NBD, the residues L390 and L391 of the linker and the residues 415–419 of the subdomain β of the SBD. These residues were identified to be crucial for the interdomain communication in DnaK. Indeed, the mutation R167A induces a loss of allosteric coupling between the NBD and the SBD [64] and the mutations K414I [90] and N415G [91] eliminate and attenuate allostery, respectively. The residues L390, L391 and L392, which belong to the VLLL motif of the linker (highly conserved between the different species of Hsp70 [67]), was shown essential for *E. coli* DnaK's *in vivo* functions [67]. In addition, it has been proposed that ATP binding to the ATPase domain causes a bending of the C-terminal part of the linker of *E. coli* DnaK at D393, in order to facilitate an insertion of the linker between the NBD and the SBD [64]. In the present MD simulations, the coupling between the NBD and the SBD confirms the insert of the linker upon ATP binding between the two domains through the interactions R167/L390 and D393/N415.

The contacts between ATP and the NBD were identified (Fig. 4 B and Fig. 4 C). This network of contacts is formed by 12 residues which belong to the subdomains IA, IIA and IIB of the NBD: T11, T12 and N13 (IA); G195, G196, G197, G198 and T199 (IIA); I271 (IIB); G341, G342 and R345 (IIA). Among these 12 residues, the CMD ATP/ADP reveals that only 6 are unique to the ATP-bound state of *E. coli* DnaK and were not found in the APO and ADP-DnaK MD simulations performed in the present work (Fig. 4 B and C and Fig. S2 C): T11, G197, G198, T199, I271 and G341.

The contacts depicted in Fig. 4 for the intermediate state ATP*-DnaK can be compared qualitatively with those observed in the very recent crystallized triple mutant ATP-bound DnaK [69]. Although the structure of the intermediate state found in MD and the crystallized open conformation of DnaK are quite different as discussed above, many contacts between ATP and the NBD found in ATP*-DnaK (Fig. 4 B and C) are also found in the experimental structure of ATP-bound DnaK (PDB ID: 4B9Q) [69]. Except D195-ATP, at a contact distance (see Methods section) of 4.9 Å in the open X-ray structure, all contacts found in MD between the NBD and ATP (Fig. 4 B) occur also in the ATP-bound experimental structure. However, contacts between ATP and the NBD observed in the fully open structure are missing in ATP* intermediate state: in particular the residue K70, which is at 2.8 Å from ATPγ in the experimental structure, is at about 4.2 Å in ATP*-DnaK. A contact occurs however between K70 and E171 both in the ATP*-DnaK MD trajectory (average distance K70-E171 = 2.4 Å) and in the experimental open conformation but not in the ADP-DnaK trajectories (average distance ADP K70-E171 = 8.6 Å). Because the metastable position of the linker is different in ATP* compared with the position of the linker in the open conformation of DnaK (Fig. 1B), different interdomain contacts were found experimentally in particular R167-D481, I168-D481, N415-D326 and D393-N170.

The analysis of the contact maps provides only a static representation of the MD data and of the influence of ATP binding on the conformations of *E. coli* DnaK. The modifications of the protein dynamics induced by the nucleotide and the correlations between the different structural modifications along the DnaK amino-acid sequence are not easily interpreted from this sole analysis. As stated in the introduction, the FEL holds the key to understand how a nucleotide modifies the properties and populations of the protein conformers. To provide the link between the FEL and the global conformational change observed in ATP-bound MD trajectories, we analyze next the free-energy profiles (FEPs) of the CGDAs *γ* (see Fig. S1 and Eq. 1) along the amino-acid sequence of *E. coli* DnaK.

### Analysis of the main-chain FEPs along the amino-acid sequence to detect key residues involved in the conformational changes

In order to identify which residues are involved in the nucleotide signal propagation along the backbone of *E. coli* DnaK, we examined which of the 597 CGDAs *γ* have a FEP depending on the nucleotide-binding state of the NBD. However, the FEPs of DnaK in a given nucleotide-binding state computed from the present unbiased MD trajectories are effective FEPs. The effective FEP differs from the actual FEP, which is an equilibrium thermodynamic property and should be computed from a large ensemble of trajectories of the protein in the same nucleotide state. However, the size of the system simulated (about 500000 atoms) and our computing capabilities limited the number of trajectories to only two trajectories of several hundred of nanoseconds per nucleotide-binding state of the NBD. Because of this limited sampling issue, only the FEPs which are similar in different trajectories in a given nucleotide-binding state were considered.

#### Convergence of the FEPs

For each *γ_i_*, the similarity index *H(γ_i_)* computed from two MD trajectories of DnaK in the same nucleotide-binding state of the NBD was calculated, i.e. *H(γ_i_)^APO1/APO2^*, *H(γ_i_)^ADP1/ADP2^* and *H(γ_i_)^ATP1/ATP2^* (see Eq. 2). For the APO state, 471 of 597 FEPs were similar (“converged”) in the two MD trajectories representing 79% of all *γ_i_* angles (defining the DATA for the APO state ≡ *{γ_i_}_APO_*). For the ADP and ATP bound states, 449 of 597 (DATA ADP ≡ *{γ_i_}_ADP_*) and 447 of 597 (DATA ATP≡*{γ_i_}_ATP_*) FEPs were similar (“converged”), respectively. The similar FEPs are located all along the amino-acid sequence, both in secondary-structures and in loops, and have various shapes from strongly harmonic FEPs to multiple-minima FEPs [71]. Only the CGDAs *γ_i_* belonging to the subset DATA for each nucleotide-binding state of the NBD of DnaK, i.e. *{γ_i_}_APO_*, *{γ_i_}_ADP_* and *{γ_i_}_ATP_*, were considered to be “converged” within the time-scale of the present MD simulations. From these DATA subsets, we defined an average PDF for each nucleotide-binding state of the NBD by combining the two MD trajectories 1 and 2 with different initial conditions i.e. *P_APO_(γ_i_)*, *P_ADP_(γ_i_)* and *P_ATP_(γ_i_)* (*P(γ_i_)≡[p_1_(γ_i_)+p_2_(γ_i_)]/2*) and recomputed the FEPs *V(γ_i_)* of each *γ_i_* of the DATA subsets from these average PDFs *P(γ_i_)*, as shown in Eq. 1.

#### Identifying a small network of residues influenced by the nucleotide binding from the FEPs

**Figure 5:**
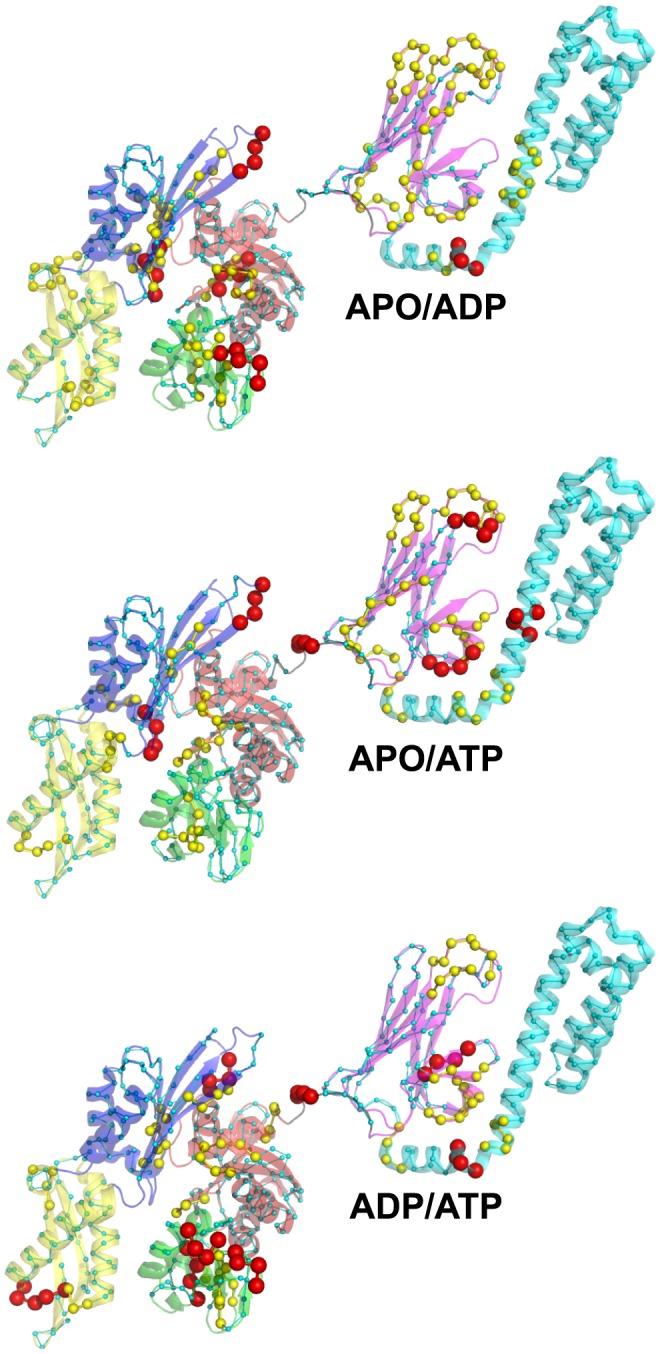
Analysis of 1-D FEPs of CGDAs *γ*. Mapping of the dissimilarity index *1-H* between the FEPs of APO/ADP (top panel), APO/ATP (middle panel) and ADP/ATP (bottom panel) onto the structure of DnaK (PDB ID: 2KHO) [70]. Residues belonging to the CGDAs *γ* which are strongly influenced (*1-H*>0.7), significantly influenced (0.3≤*1-H*<0.7) and weakly influenced by the nucleotide (*1-H*≤0.3) are shown with large red, medium yellow and small cyan spheres, respectively. The color code of the protein structure is the same as in Fig. 1. These figures were prepared with PyMOL [http://www.pymol.org].

To identify the key residues influenced by the nucleotide-binding state of the NBD, the *dissimilarity* index, defined by *1-H(γ_i_)* (Eq. 2), was computed between the average FEPs (see above) *V_APO_(γ_i_) V_ADP_(γ_i_)*, and *V_ATP_(γ_i_)*, for the CGDAs common to the different DATA sets (388 *γ_i_* for *{γ_i_}_APO_*∩*{γ_i_}_ADP_*, 379 *γ_i_* for *{γ_i_}_APO_∩{γ_i_}_ATP_* and 363 *γ_i_* for *{γ_i_}_ADP_*∩*{γ_i_}_ATP)_*). The results for the dissimilarity *1-H(γ_i_)* along the amino-acid sequence are shown in Fig. 5 (on the structure) and in Fig. S3 (along the sequence). For each comparison, APO/ADP, APO/ATP and ADP/ATP, the FEPs were divided in three sets according to the influence of the nucleotide-binding state of the NBD of DnaK: a set of FEPs strongly influenced (*1-H*>0.7), a set of FEPs significantly influenced (0.3≤*1-H*<0.7) and a set of FEPs weakly influenced (*1-H*≤0.3) by the nucleotide. Most of CGDAs *γ* of DnaK have FEPs weakly influenced by the nucleotide-binding state of the NBD (more than 300 in the three comparisons, see Fig. 5, Fig. S3, and Table 2) showing that the large conformational changes induced by a nucleotide could be due to a small network of residues. For example, only 9 FEPs, which are distributed all along the structure (Fig. 5) and along the amino-acid sequence (Fig. S3), are strongly influenced by the nucleotide between the ADP and the ATP-bound states of DnaK (*1-H*>0.7) and 25 FEPs are significantly influenced (0.3≤*1-H*<0.7). Similar results were observed for the other comparisons (Fig. 5, Fig. S3, and Table 2).

**Table 2:** Statistical analysis of the dissimilarity [*1-H(γ_i_)*] of the FEPs between concatenated MD runs in different nucleotide-binding states.

Subset	APO/ADP	APO/ATP	ADP/ATP
**Strongly influenced**	{6}/{388} (1.6%)	{6}/{379} (1.6%)	{9}/{363} (2.5%)
**Significantly influenced**	{41}/{388} (10.6%)	{26}/{379} (6.9%)	{25}/{363} (6.9%)
**Weakly influenced**	{341}/{388} (87.8%)	{347}/{379} (91.5%)	{329}/{363} (90.6%)

*1-H(γ_i_)*>0.7, 0.3<*1-H(γ_i_)*≤0.7 and *1-H(γ_i_)*≤0.3, respectively. The notation is the following: {number of residues in the subset}/{number of residues compared}. Subset strongly, significantly and weakly influenced correspond to

**Figure 6:**
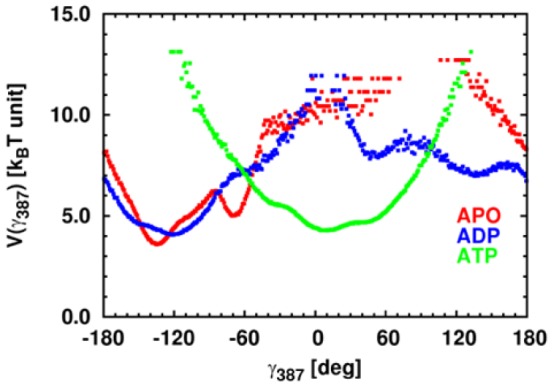
FEPs *V(γ)* for *γ_387_* in *k_B_T* unit. *V(γ)* computed from APO, ADP-bound and ATP-bound MD simulations of DnaK are colored in red, blue and green respectively.

The combination of the three different comparisons reveals common CGDAs *γ* for which the FEP is significantly influenced by the nucleotide-binding state of the NBD (Fig. S3). For example, *γ_387_* in the linker has a FEP weakly influenced by the nucleotide in the comparison APO/ADP (*1-H(γ_387_)^APO/ADP^* = 0.26) and strongly influenced in the comparisons APO/ATP (*1-H(γ_387_)^APO/ATP^* = 0.99) and ADP/ATP (*1-H(γ_387_)^ADP/ATP^* = 0.98) (Fig. S3). It means that *γ_387_* has a specific FEP in ADP-bound DnaK or in nucleotide-free DnaK but another FEP in ATP-bound DnaK (Fig. 6). We named hereafter *γ_387_* an ATP-specific angle. Another example is *γ_98_* which has a FEP weakly influenced by the nucleotide in the comparison APO/ATP (*1-H(γ_98_)^APO/ATP^* = 0.18) but strongly influenced in the comparisons APO/ADP (*1-H(γ_98_)^APO/ADP^* = 0.75) and ADP/ATP (*1-H(γ_98_)^ADP/ATP^* = 0.78), as shown in Fig. S3. It means that *γ_98_* has a specific FEP in ATP-bound DnaK or in nucleotide-free DnaK but another specific FEP in ADP-bound DnaK (Fig. S4). We named hereafter *γ_98_* an ADP-specific angle.

**Figure 7:**
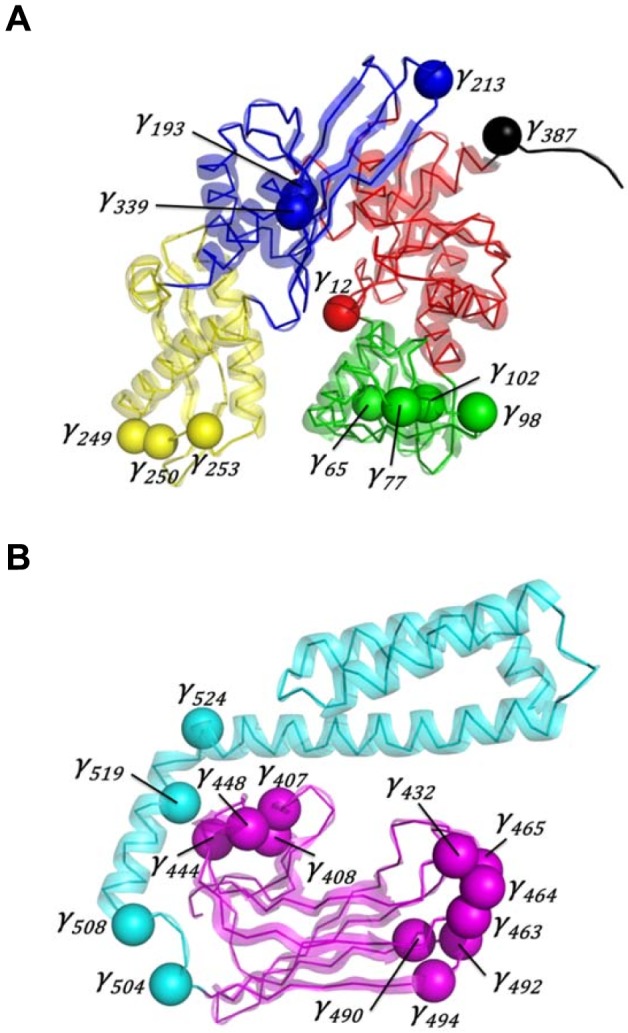
Overview of the 27 CGDAs *γ*. A: NBD+linker. B: SBD. Each CGDA *γ_i_* is represented by a sphere centered on the C^α^(i) atom. The color code of the protein structure is the same as in Fig. 1. These figures were prepared with PyMOL [http://www.pymol.org].

Finally, 7 CGDAs *γ* are APO specific (for example *γ_213_*, Fig. S4), 6 CGDAs *γ* are ADP specific, 9 CGDAs *γ* are ATP specific and 5 CGDAs *γ* are specific to each different nucleotide-bound state (for example *γ_465_*, Fig. S4). These 27 CGDAs *γ* (Fig. 7 and Table 3) correspond to 91 residues (15% of the total residue number) which contribute to the communication between the SBD and the NBD of DnaK through nucleotide binding in the present MD simulations. The 27 CGDAs *γ* are localized along the whole structure, as shown in Fig. 7, and more precisely, 11 are located in the NBD (1 in the subdomain IA; 4 in the subdomain IB; 3 in each subdomain IIA and IIB), 1 in the linker and 15 in the SBD (11 in the SBD-β and 4 in the SBD-α).

**Table 3:** List of the 27 CGDAs *γ* revealed by analysis of 1-D FEPs of DnaK in different nucleotide-binding states and their corresponding amino acids.

			*H(γ_i_)*			
CGDA *γ_i_*	Corresponding residues	Location	APO/ADP	APO/ATP	ADP/ATP	Refs.
213†	G212-E213-K214-T215	IIA	**0.01**	**0.19**	0.86	[12], [25], [65]
432† ^,^ ‡	D431-N432-Q433-S434	SBD-β	**0.54**	**0.53**	1.00	[91], [98], [100]
463† ^,^ ‡	I462-N463-P464-A465	SBD-β	**0.45**	**0.64**	0.94	[91], [102]
490	K489-D490-K491-N492	SBD-β	**0.49**	**0.36**	0.95	
492	K491-N492-S493-G494	SBD-β	**0.57**	**0.62**	0.93	
494‡	S493-G494-K495-E496	SBD-β	**0.64**	**0.53**	0.97	[91]
504	A503-S504-S505-G506	SBD-α	**0.41**	**0.64**	0.73	
77†	R76-F77-Q78-D79	IB	**0.32**	0.87	**0.16**	[89], [96]
98	D97-N98-G99-D100	IB	**0.25**	0.82	**0.22**	
102†	A101-W102-V103-E104	IB	**0.35**	0.75	**0.61**	[97]
193‡	V192-Y193-D194-L195	IIA	**0.63**	0.99	**0.66**	[25], [91]
253†	L252-R253-N254-D255	IIB	**0.63**	0.99	**0.70**	[12], [99]
339	I338-L339-V340-G341	IIA	**0.46**	0.98	**0.34**	
12† ^,^ ‡	T11-T12-N13-S14	IA	0.99	**0.46**	**0.40**	[91], [95]
249†	Q248-G249-I250-D251	IIB	0.89	**0.48**	**0.28**	[12], [99]
250†	G249-I250-D251-L252	IIB	0.95	**0.37**	**0.26**	[12], [99]
387†	V386-K387-D388-V389	linker	0.74	**0.01**	**0.02**	[65], [67], [69], [98]
407† ^,^ ‡	G406-V407-M408-T409	SBD-β	0.87	**0.43**	**0.43**	[91], [100]
408†	V407-M408-T409-T410	SBD-β	0.79	**0.52**	**0.62**	[100]
448† ^,^ ‡	R447-A448-A449-D450	SBD-β	0.87	**0.67**	**0.54**	[91], [98], [103]
508	L507-N508-E509-D510	SBD-α	0.98	**0.67**	**0.59**	
524†	A523-E524-A525-D526	SBD-α	0.98	**0.56**	**0.59**	[103]
65†	N64-T65-L66-F67	IB	**0.53**	**0.45**	**0.12**	[89], [96]
444† ^,^ ‡	G443-E444-R445-K446	SBD-β	**0.64**	**0.19**	**0.68**	[91], [100]
464‡	N463-P464-A465-P466	SBD-β	**0.49**	**0.49**	**0.56**	[91]
465‡	P464-A465-P466-R467	SBD-β	**0.65**	**0.50**	**0.63**	[91]
519‡	D518-A519-E520-A521	SBD-α	**0.22**	**0.65**	**0.05**	[91], [102], [103]

† and ‡ indicate amino acids which have been depicted to be allosterically relevant from biochemical and bioinformatics methods, respectively. Characters in bold in the table represents strongly and significantly influenced FEPs (*H*<0.7, see Table 2 and text for more details).

### Two-dimensional free-energy surface (FES) built from the collective motions of the key residues in ATP-bound DnaK

#### Two-dimensional FES of ATP-bound DnaK

The comparison of the FEPs of CGDAs *γ* of the backbone of DnaK in different nucleotide-binding states reveals that the nucleotide influences at least 27 CGDAs *γ* located both in the NBD and in the SBD (Fig. 7). In other words, the modification of the (unknown) FEL of DnaK upon ligand binding [4], [7] can be represented here quantitatively by its projection over 27 dimensions. Obviously, these 27 coordinates are not independent and the local motions of the CGDAs *γ* upon nucleotide-binding are coupled to each other. In order to understand how the binding of ATP to the NBD of DnaK induces the docking of its SBD onto its NBD, we established the relation between these 27 local motions *γ* and the global motions (domains) of ATP-DnaK by applying dPCA [74].

The dPCA was carried on the subset of 27 CGDAs *γ* using the MD trajectories of ATP-DnaK (see Methods section for more details). Results for the MD trajectory ATP1 are shown (the results for the MD trajectory ATP2 lead to the same conclusions [92]). Only modes 1 and 2 contribute significantly to the *MSF* of the 27 CGDAs. Indeed, for the MD run ATP1, *λ^1^* contributes 74% of the total covariance of the system and *λ^2^* 8% whereas for the MD run ATP2, *λ^1^* contributes 85% of the total covariance of the system and *λ^2^* 3%; justifying to use only the two collective modes 1 and 2 as the minimal representation of the free-energy landscape of ATP-DnaK.

**Figure 8:**
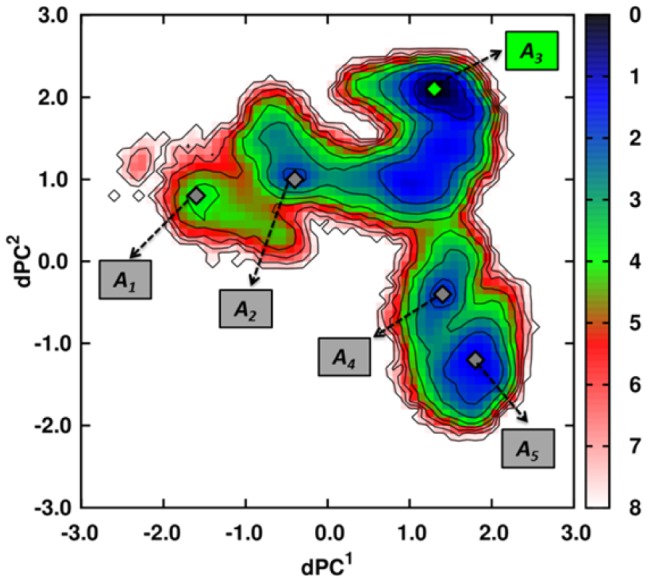
Dihedral principal component analysis applied to MD simulations of ATP-bound DnaK. FES computed for the MD run ATP1. Minima are shown with gray (green for the most probable one) diamonds and the isolines (black lines) are drawn every *k_B_T* unit. The color scale for the free-energy is in *k_B_T* units.

The projections of the vectors *u_n_(t)* (Eq. 4) extracted from the MD trajectory along the eigenvectors of modes 1 and 2 defined the collective coordinates *dPC^1^(t)* and *dPC^2^(t)*, respectively (Eq. 7). From the two-dimensional PDF of *dPC^1^* and *dPC^2^* computed from the MD trajectory, i.e. *P(dPC^1^_,_dPC^2^)*, the effective FES *V_1,2_ = -k_B_T ln[P(dPC^1^_,_dPC^2^)]*, shown in Fig. 8, was calculated. The largest motions defined by the first and second *dPCs* can be interpreted in terms of specific features of the two-dimensional FES *V_1,2_* because the dPCA method is converged *in the subspace* of the 27 dihedral angles considered (see Methods section and Refs. [93] and [94]). The FES of the MD run ATP1 shows 5 global minima *A_i_* (Fig. 8) defined from the isolines of free-energy (≤3 *k_B_T*). The minima *A_i_* are ordered according to the chronology of the MD trajectory, from *i* = 1 to 5. The most probable structure within each minimum *A_i_* of the MD run ATP1 is shown in Fig. S5: the structure corresponding to the minimum *A_1_* is an elongated structure, without interactions between the NBD and the SBD and closely similar to the initial one (PDB ID: 2KHO) [70]. In the basin corresponding to the minimum ***A_2_***, the SBD rotates around the linker and get close to the NBD but without strong interactions between the two main domains. In the structure of the global minimum ***A_3_*** of the MD run ATP1, the SBD is docked to the lobe I of the NBD (Fig. S5). Note that the lid of the SBD (SBD-α) is docked in the subdomain B of the lobe I of the NBD. Structures corresponding to the minima ***A_4_*** and ***A_5_***, are characterized by the docking of the two domains, as observed in the minimum ***A_3_***, but with local conformational changes in the SBD (for example of the CGDA *γ_504_*, Fig. S6).

In the representative structure of the global minimum of the MD run ATP2, as shown in Fig. 2, the SBD is docked to the lobe I of the NBD but in a different position, compared to the global minimum of the MD run ATP1. Although the two end points of the ATP-bound DnaK trajectories are different, the ground-state (on the time-scale of our simulations) is identical in the space of the subset of CGDAs with a similar FEP. Indeed, each of the 27 CGDAs *γ* is in the same local conformation in the structures corresponding to the global minimum of the FESs computed from the MD runs ATP1 and ATP2, as shown in Fig. S6.

Because the dPCA catches the most important fluctuations occurring in a MD trajectory, the application of the dPCA analysis to the APO and ADP-bound DnaK trajectories produce different behaviors. The FES of the ADP-bound DnaK trajectories (computed from the *dPC^1^* and *dPC^2^* on the 27 dihedral angles) shown no transition state between the initial and final conformations of the protein [92]. The FES of the nucleotide-free (APO) DnaK trajectories (computed from the *dPC^1^* and *dPC^1^* on the 27 dihedral angles) shown three basins: a small basin corresponding to the initial state, a transition state, and a final basin corresponding to the final state [92]. The transition state corresponds to an intermediate state where the SBD starts to rotate on a cone due to a change of the linker conformation [92].

#### Free-minimum energy path on the two-dimensional FES of ATP-bound DnaK

**Figure 9:**
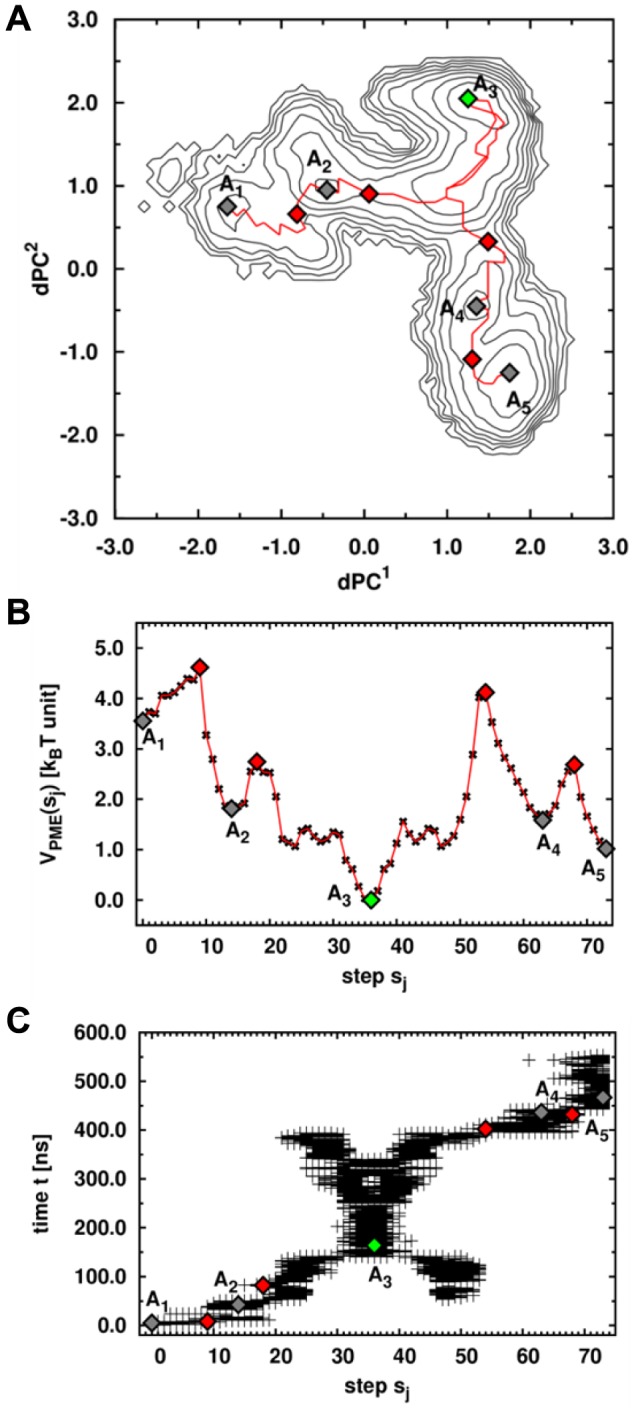
Analysis of the FEL of ATP-bound DnaK. A: FES generated from dPCA of MD run ATP1. Minima and saddle points are shown with gray (green for the most probable one) and red diamonds, respectively. The PME is represented by the red line. B: Free-energy *V^PME^* along the PME for the transition ***A_1_→A_2_→A_3_→A_4_→A_5_***. C: Results of the clustering along the PME. Each frame is represented at the corresponding time *t* observed in the MD trajectory.

The initial ***A_1_*** and final ***A_5_*** conformations are located at the bottom of two “basins” of the FES (Fig. 8) which can be connected by a pathway of minimum energy (PME) (Fig. 9 A). The PME for the transitions ***A_1_→A_2_→A_3_→A_4_→A_5_*** on the FES of the MD run ATP1 was computed as described in the Methods section. The PME was divided into 74 steps (from *s_0_* to *s_73_*) with minima ***A_1_***, ***A_2_***, ***A_3_***, ***A_4_*** and ***A_5_*** at respectively *s_0_*, *s_14_*, *s_36_*, *s_63_* and *s_73_* (Fig. 9 A and B). The PME shows 4 saddle points for each transition: at *s_9_* for the transition ***A_1_→A_2_***; at *s_18_* for the transition ***A_2_→A_3_***; at *s_54_* for the transition ***A_3_→A_4_*** and at *s_68_* for the transition ***A_4_→A_5_***. The largest free-energy barrier along this pathway is observed for the transition ***A_3_→A_4_*** (Fig. 9 B) and is larger than 4.0 *k_B_T* (≈2.5 *kcal/mol*).

From the FES (Fig. 9 A), we extracted the coordinates of the bin (*dPC^1^*; *dPC^2^*) of each step *s_j_* along the PME. Each step *s_j_* of the PME, which is a bin of the FES (see Methods section), represents a number *N(s_j_)* of frames of the corresponding MD trajectory. For example, the step *s_1_* corresponds to the bin (*dPC^1^* = −1.53; *dPC^2^* = 0.64) and to *N(s_1_)* = 87 frames of the MD run ATP1. From the *N(s_j_)* frames, we computed the 1-D FEPs *V_j_(γ_i_)* of the 27 CGDAs *γ* used in the dPCA, and we defined the value *γ_i_(s_j_)* as the minimum of the FEP *V_j_(γ_i_)* (the most probable value of the CGDAs *γ_i_* at each step *s_j_*). Finally, for each of the 27 CGDAs *γ_i_*, we projected the trajectory *γ_i_(s_j_)* along the PME and along its corresponding one-dimensional FEP *V(γ_i_)* (shown in Fig. S6) to visualize the coupling between the local and the global collective motions described by *dPC^1^* and *dPC^2^* (as shown in Fig. S7). The CGDAs *γ* with the largest changes along the PME are the CGDAs having multiple-minima FEPs (except for *γ_387_*) (Fig. S7), corresponding also to those having a significant influence (see Eq. 5 and Fig. S8) in the *dPC* modes [*Δ_i_^1^* (mode 1) and *Δ_i_^2^* (mode 2)>10%], i.e. *γ_77_*, *γ_98_*, *γ_213_*, *γ_387_*, *γ_407_*, *γ_464_*, *γ_504_* and *γ_519_*. In addition, others CGDAs *γ*_i_ show smaller changes along the PME, for example *γ_250_*, *γ_432_*, *γ_444_*, *γ_465_* or *γ_492_*. In total, these 13 *γ*_i_ CGDAs correspond to 49 residues.

At *s_0_*, corresponding to the minimum *A_1_* on the FES (Fig. 8), the structure is still elongated and without interactions between the NBD and the SBD and is closely similar to the initial structure (Fig. S9). From *s_0_* to *s_9_* (corresponding to the saddle point of the transition *A_1_→A_2_*), we observed the rotation of the SBD around the linker (Fig. S9), due to the relaxation of *γ_98_*, *γ_465_* and *γ_492_* towards the minimum of their FEPs (Fig. S7), involving a reorganization of subdomains IB of the NBD and of the SBD-β (Fig. S9). The system goes through this saddle point from *s_9_* to *s_14_* as a consequence of jumps of *γ_213_*, *γ_387_* and *γ_432_* to local minima of their FEPs and last but not least *γ_504_* jumps to its most probable conformation (Fig. S7). These changes involve a large rotation of the SBD of ATP-DnaK around the linker, which becomes close to the NBD (Fig. S9) at *s_14_*, corresponding to the minimum *A_2_*. The rotation of the SBD is thus related to the relaxation of dihedral angles *γ_98_*, *γ_465_* and *γ_492_* involving a reorganization of subdomains IB of the NBD and of the SBD-β (Fig. S9). The angle *γ_98_* is located within the NBD and coupled to the 27 other dihedral angles, in particular to *γ_12_* built on the residues T11, T12, N13 which are in contact with ATP (Fig. 4) providing a physical link between the SBD rotation and the ATP/NBD interactions. For the transition *A_2_→A_3_* (from *s_14_* to *s_36_*), *γ_387_*, *γ_444_* and *γ_519_* change their conformation (Fig. S7), allowing the system to go through the saddle point at *s_18_*, which involves a rotation of the subdomain SBD-α of ATP-DnaK, which becomes close to subdomain IB of the NBD (Fig. S9). Then, the system relaxed to a state where the SBD is finally docked to the lobe I of the NBD (Fig. S9) at *s_36_* with changes of *γ_77_*, *γ_213_*, *γ_250_*, *γ_387_* and *γ_432_* (Fig. S7), and adopted its most stable structure in the MD trajectory ATP1, corresponding to the global minimum *A_3_* of the FES (Fig. 8).

In the MD trajectory ATP1 (on the contrary to trajectory ATP2, data not shown), the system did not end in its most stable state (*A_3_*) but escaped from this minimum because of changes of *γ_213_*, *γ_464_* and *γ_465_* which jumps to local metastable states of their FEPs, which allows the system to climb the highest energy barrier (4 *k_B_T*) along the PME at *s_54_* (Fig. 9 B). The system goes through this saddle point of the transition *A_3_→A_4_* at *s_54_* with changes of *γ_98_* and *γ_407_* (Fig. S7) which jump to local minima of their FEPs and the system relaxed to the minimum *A_4_*. The structure corresponding to the minimum *A_4_* is still characterized by a docking of the two domains (Fig. S9) but with local changes in the SBD, particularly in the SBD-β. Finally, the last transition which occurred is the transition *A_4_→A_5_*, with a saddle point at *s_68_* (Fig. 9 B). The system goes through the saddle point by a change of *γ_407_* coupled with a change of *γ_504_* up to the second most probable state along the PME, namely *A_5_* (Fig. S7). The structure in *A_5_* is characterized by the docking of the SBD on the NBD but with structural changes within the SBD and particularly within the SBD-β compared with the representative structure of the minimum *A_3_* (Fig. S9). Combination of the global (*dPC*) and local (*γ*) coordinates allow us to decipher the mechanistic process by which the protein undergoes a conformational change upon ATP binding along the PME.

Another point to figure out is the relation between the PME on the two-dimensional FES and the MD trajectory or in other terms, the relation between the step *s_j_* of the PME and the time *t* of the different events observed in the MD trajectory. As explained in Methods section, each step *s_j_* of the PME is characterized by a number of frames *N(s_j_)* of the MD trajectory. So we extracted and plotted the time *t* for each frame *i*, *i* = 1 to *N(s_j_)* (Fig. 9 C). Starting from the minimum *A_1_*, the MD trajectory reaches the docked state, which corresponds to the minimum *A_3_* after 150 ns. The basin corresponding to this minimum is explored during the major time period of the MD trajectory (up to 400 ns). The “X” shaped of the graph during this period (between 150 and 400 ns) shows us that it exists different attempts to escape this minimum *A_3_* (there are in fact three entrances/exits into/from this state) but this only happens when all the conditions given previously in the text about local conformational changes are reached (at *t* = 400 ns). As shown in Fig. 9 A, it is due to the existence of the highest barrier between the minima *A_3_* and *A_4_*. Finally, the system reaches the minimum *A_5_* at *t* = 500 ns and stay in the corresponding basin up to the end of the trajectory.

## Discussion

The analysis of the FEPs, as shown in the previous section, revealed the 27 CGDAs *γ* of *E. coli* DnaK influenced by the nucleotide-binding state of the NBD (Table 3). The 91 residues associated to these 27 CGDAs *γ* (Table 3) are involved in the long-range (on about 5–10 nm) propagation of the conformational fluctuations between the NBD and the SBD of *E. coli* DnaK. Starting from *γ_12_* in the NBD, which corresponds to residues in direct contacts with the ATP nucleotide, the signal induced by the nucleotide binding is propagated up to *γ_524_*, in the SBD, through *γ_387_*, located in the inter-domain linker (Fig. 7). Experimentally, X-ray crystallography, NMR spectroscopy, biophysical and biochemical techniques have contributed to understand such an allosteric communication in Hsp70 s (Table 3). A large comparison between the residues elucidated from the analysis of the FEPs similarity computed from the MD simulations (Table 3) and the experimental database of key residues relevant for the communication in DnaK available in the literature, shows that most of the residues identified by our methodology have been found to be experimentally relevant for the allosteric communication in Hsp70 s and that new residues can be proposed to play a role according to the present MD calculations.

In more detail, the comparison in Table 3 can be summarized as follows. In the NBD, mutation of residue T13 in bovine Hsc70 (T11 in *E. coli* DnaK) to S13 completely abrogated the communication between the NBD and the SBD [95]. Residue T13 was indeed found from the analysis of the FEP of *γ_12_*, which belongs to the nucleotide-binding pocket (Fig. 7). Mutation in bHsc70 of residue K71 (K70 in *E. coli* DnaK), which is the only residue of subdomain IB in contact with the ATP molecule, affects the ATP hydrolysis [96] and also abrogated the ATP-induced conformational changes determined by SAXS experiments [89]. In the network extracted from our analysis, several CGDAs *γ*, having a significant role for signal propagation, belong to subdomain IB of the NBD and particularly *γ_65_* and *γ_77_*, which enclosed residue K70 (Fig. 7). In addition, fluorescence of W102, the only tryptophan residue of *E. coli* DnaK [97] is directly dependent on the ADP/ATP conformational changes in the NBD and was identified from the analysis of the FEP of *γ_102_* in the present MD simulations. In the NBD, the interface between the subdomains IA and IIA plays a key role in Hsp70, acting as a cleft for the binding of the interdomain linker [12], [65], [86] in the ATP-bound state (open structural state) of Hsp70 [65]. The analysis of the FEPs of DnaK reveals the important role of CGDA *γ_213_*, which is precisely localized in subdomain IIA, and of the CGDA *γ_387_*, which is part of the linker (Fig. 7).

The linker is the physical link between the NBD and the SBD and was identified early as a key region for the allosteric communication in Hsp70 s [64], [65], [67], [98]. The linker is flexible and solvent exposed in the nucleotide-free and in the ADP-bound DnaK and is docked in the IA/IIA cleft of the NBD in the ATP-bound state. In the present MD simulations, the CGDA *γ_387_* belonging to the linker occurs in a specific conformation in ATP-DnaK (Fig. 6), confirming that ATP binding lead to a conformational change in the linker region (even if the SBD stays closed in the present ATP-bound DnaK trajectories).

Finally, the dynamics of the subdomain IIB of the NBD, and particularly its rotation, is known to be allosterically relevant [12], [66], [69], [99]. The rotation of the subdomain IIB occurs upon nucleotide exchange from ADP to ATP and the binding of ATP involves the opening of the nucleotide-binding cleft relative to an ADP-bound NBD. These findings are confirmed by our analysis of the FEPs which revealed that *γ_249_*, *γ_250_* and *γ_253_* are influenced by the nucleotide; these CGDAs *γ* are localized in the loop between two α-helices of the subdomain IIB of the NBD for which large changes in the NMR chemical-shifts between the ADP-bound and the ATP-bound NBD of *E. coli* DnaK were observed [12]

Most of the CGDAs *γ* located in the SBD of DnaK influenced by the nucleotide-binding state of the NBD are located in the SBD-β subdomain (Fig. 7). These CGDAs are related to residues previously identified experimentally. Indeed, *γ_408_*, *γ_432_* and *γ_444_* involve residues M408, D431 and E444 which modify the substrate binding and affinity to *E. coli* DnaK [100], [101]. In addition, residue I462, belonging to *γ_463_*, has been shown to be allosterically relevant due to the fact that the dual DnaJ and peptide-binding defects were observed in I462T [102]. In addition, amide hydrogen exchange experiments [98] revealed that the binding of ATP in the NBD of Hsp70 involves local structural changes in the segments 413–437 (which encloses the CGDA *γ_432_* found in the analysis of the FEPs) and 439–457 (which encloses the CGDA *γ_448_* found in the analysis of the FEPs) of *E. coli* DnaK (Fig. 7).

Other CGDAs *γ* revealed by our analysis (see Table 3 and Fig. 7) such as *γ_98_*, *γ_339_*, located in the NBD, *γ_490_* and *γ_492_*, located in the SBD-β, or *γ_504_*, *γ_508_* and *γ_524_* located in the SBD-α provide new hypothesis for the residues relevant for the allosteric communication in Hsp70. Interestingly, the residue N98 contacts G506 in the crystal form of ATP-bound DnaK [69]. The mutation V337F, a residue preceding the residues defining *γ_339_* strongly reduces the capacity of DnaK to refold luciferase [103]. Finally, *γ_524_* includes the residue D526 forming a highly conserved ionic pair with R445 which stabilizes the SBD-α/SBD-β interactions through an ionic network including also K446 (precedes *γ_448_*, Table 3), D518 (belonging to *γ_519_*, Table 3) and D450 (belonging to *γ_448_*, Table 3) [104]. Two GCDAs *γ* are located at the interface between the SBD-β and the SBD-α, namely *γ_504_* and *γ_508_* and one is located in the kink between the helices A and B of the SBD-α, namely *γ_524_* (Fig. 7). This demonstrates that even if the SBD remains closed in ATP*-DnaK, local conformational changes within the SBD were observed in MD.

**Figure 10:**
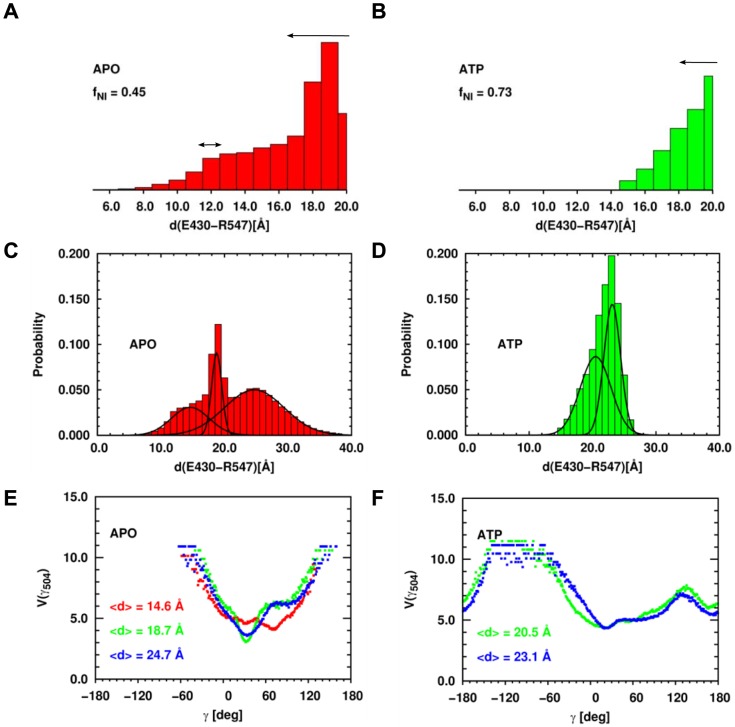
Comparison between MD data and EPR experiments. Histograms of the distance E430-R547 computed from the MD simulations in the APO state (panels A and C, red) and in the ATP-bound state (panels B and D, green). Panels A and B represent the distributions up to 20 Å, as in the EPR experiments and panels C and D represents the same distributions up to 40 Å. Each subpopulation in the distance distribution was fitted with a Gaussian function shown with black lines. The black arrows in panels A and B represent the experimental results from Ref. [81]. FEP *V(γ_504_)* computed from different subpopulations relative to the distance E430/R547 distribution function in the APO state (panel E) and in the ATP state (panel F). *<d>* represents the mean value of the Gaussian distributions shown in panel C for the APO-DnaK and D for the ATP-DnaK.

The conformational changes of the SBD induced by ATP binding in MD can be related to recent experimental data. Indeed, EPR spectroscopy [81] has been recently used to investigate the conformations of the SBD-α relative to those of the SBD-β of *E. coli* DnaK. Distance distributions between the residues E430 and R547 of the nucleotide-free DnaK and of ATP-bound DnaK were extracted from the EPR spectra (Fig. 10). The E430/R547 distance distribution measured in nucleotide-free DnaK shows two interacting spin populations, one around 12 Å and one with a broader population between 16 Å and 20 Å (Fig. 10 A, black arrows). Only a small fraction of the labeled residues was not interacting (fraction of non-interacting spins, *f_NI_* = 0.09), corresponding to residues distant by more than 20 Å. Upon ATP binding, the short-distance population disappeared and almost 80% of the spin labeled residues was separated by more than 20 Å, whereas in 20% of the molecules, the distance was in a range similar to the nucleotide-free state (Fig. 10 C and D, black arrows).

To compare with EPR data [81], we computed the probability distribution of the distance between the centers of mass of the side chains of E430 and R547 from the MD trajectories of APO-DnaK and ATP-DnaK. Up to 20 Å, the distance distribution of nucleotide-free DnaK shows two populations; a broad distribution centered at 14.6 Å and a narrower distribution centered at 18.7 Å (Fig. 10 A). Note that the MD simulations provide information on the E430/R547 distance distribution for distances inaccessible to EPR (>20 Å): in the case of the nucleotide-free DnaK, a third population is observed and is characterized by a broad distribution centered at 24.7 Å (Fig. 10 C). The fraction of distances larger than 20 Å in MD of the nucleotide-free DnaK (*f_NI_*) is about 45%. Upon ATP binding, the population of the shortest distance observed in the nucleotide-free DnaK (centered at 14 Å) disappears (Fig. 10 B), as observed in EPR experiments, and the distance distribution shows two peaks centered at 20.5 Å and 23.1 Å, respectively (Fig. 10 D). Consequently, the fraction of distances larger than 20 Å increases up to 73%, as observed in EPR (Fig. 10 C and D). These MD results show that the addition of ATP increases the distances between the SBD-β and the SBD-α even if the SBD remains closed. In addition, the MD simulations provide a possible structural origin for the disappearance of the population of molecules with the shortest E430/R547 distance upon ATP-binding in the experiment. Indeed, we performed a clustering of the *E. coli* DnaK structures within each subpopulation of the distance distributions shown in Fig. 10 B and D and computed the FEPs of each of the CGDA *γ* selected previously and which belong to the SBD (see Table 3). From these calculations, we found that *only* the FEP of *γ_504_* was different in each subpopulation of molecules having a different E430/R547 distance (Fig. 10 E and F). In the APO state, the structures belonging to the shortest distance population have a most probable value of *γ_504_* equal to 71.5° whereas it is 55.5° for the structures in the 2 largest subpopulations (Fig. 10 E). In the ATP state, *γ_504_* moves more freely compared with the APO state (Fig. 10 F), meaning that the lid is more mobile in this nucleotide-binding state compared with the nucleotide-free state, which could explain that the shortest distance population distribution observed for the nucleotide-free DnaK disappears for the ATP-bound DnaK.

A statistical analysis of 926 Hsp70 sequences pointed to interdomain sectors that might mediate the allosteric communication between the NBD and the SBD of Hsp70 [91]. Although it is unclear that the allosteric mechanism and residues can be extracted from the sole comparison of sequences [60], we found that these sectors correspond to key CGDAs found from the present analysis of the FEL of DnaK. The sectors comprise the residues 11, 12 and 14 (*γ_12_*); residues 192 and 195 (*γ_193_*); residue 406 (*γ_407_*); residues 431 and 433 (*γ_432_*); residues 443 to 445 (*γ_444_*); residue 450 (*γ_448_*); residues 462, 463, 464, 466 and 467 (*γ_463_*, *γ_464_* and *γ_465_*); residue 494 (*γ_494_*) and finally residue 519 (*γ_519_*) (where the CGDAs in the brackets are shown in Table 3). In total, 19 residues (involved in 10 CGDAs) out of 91 residues (involved in 27 CGDAs) revealed by the present MD simulations were also found by a statistical analysis of Hsp70 sequences [91]. Due to the importance and relevance of hHsp70 in diseases, it is instructive to examine to which extent the residues identified in the allosteric mechanism of DnaK by MD are conserved in hHsp70. In order to answer this question, we performed the alignment of the sequence of *E. coli* Hsp70 (Uniprot ID: P0A6Y8) on the sequence of the human inducible Hsp70 (Uniprot ID: P08107) (Table S1). On the 91 residues of *E. coli* Hsp70 found to be relevant in the nucleotide-induced conformational change, 59/91 [65%] are positives [with similar physical and chemical properties] among which 44/91 [48%] are identical, i.e. conserved in the human form. Only 2/91 are gaps [2%]. In addition, at least one residue of each CGDA found to be dependent on the nucleotide-binding state of the NBD of DnaK is common between human and the *E. coli* Hsp70 s. However, one emphasizes that the conservation of the residues involved in the conformational changes of DnaK do not necessarily guarantee that the allosteric mechanisms of DnaK and hHsp70 are similar [60].

Residues involved in the dynamics of the conformational change of DnaK are also largely conserved in hHsp70. In DnaK, 49 residues are involved in the collective modes 1 and 2 computed from the dPCA of the MD trajectories as identified by their influence (Eq. 5) *Δ_i_^1^* (mode 1) and *Δ_i_^2^* (mode 2) (Fig. S8) and by their dynamics along the PME (Fig. 9). To identify these residues in hHsp70, we aligned the sequences of DnaK and hHsp70 (Table S1) and reported the results of the alignment in Table 4. On 49 residues identified in this manner, 2 are gaps and 33 are positives (Table 4). As stated in the introduction, the low-frequency vibrational modes of proteins are generally correlated with the reaction coordinates of their conformational changes [49]. Based on all-atom normal modes of hHsp70 [23], [49], we identified 64 residues having the largest influences in the low-frequency vibrational modes [92] of hHsp70 which are compared to those deduced from dPCA in Table 4. Comparison between the NMA and dPCA shows that 12 residues are identical in both methods, namely F78, K100, K250, K251, G408, V409, A467, P468, R469, K493, S494 and T495 (Table 4). The most important differences between the two approaches were observed in the subdomain IIA of the NBD, in the linker and in the SBD-α where we did not find residues common to the two analyses. The differences observed are expected because NMA describes the structural fluctuations of the protein in the vicinity of the minimum of the free-energy basin characterizing its native state (harmonic approximation) whereas the dPCA analysis include jumps between minima of the protein FEL.

**Table 4:** List of residues found to be relevant for conformational dynamics of *human* Hsp70 deduced from NMA of human Hsp70 [49], [92] and from dPCA of *E. coli* MD trajectories (present work) after sequence alignment of *E. coli* DnaK on human Hsp70 (Table S1).

Location	NMA	dPCA
NBD IA		
NBD IB	L50- **F78**-***K100***-*Y107*-K108-E110-*K112*	K77-**F78**-*G79*-D80-D99-***K100***-*P101-K102*
NBD IIA	R193	G215-*I216*
NBD IIB	R247-*H249*-***K250*** **-** ***K251***-*S254*-*Q255*-R299	***K250***-***K251***-D252-I253
linker		V388-Q389-D390-L391
SBD-β	**G408-V409**-T429-*Y431*-*S432*-*Q441*-*S462*-I464-**A467**-**P468**-**R469**-G470-V471-P472-Q473-D492-**K493**-**S494**-**T495**-K497-*A498*-N499-K500	**G408**-**V409**-M410-T411-D433-N434-Q435-*P436*-G445-E446-R447-*A448*-*P465*-P466-**A467**-**A468**-**R469**-**K493-S494-T495**-G496
SBD-α	*Y525*-*K526*-*Q532-R533*-*R535-Y545-K559*-K561-*S563-E564-A565*-D566-K567-*K568-K569-K573*-*W580*-E588-K589-*D590*-*E591-F592-H594-R596*- K597-Q601	*N505-D506-K507*-G508-E521-A522-E523-*K524*

Residues common to the two analyses are in bold. Residues of hHsp70 which are not identical in *E. coli* Hsp70 are in italic font.

### Conclusion

In summary, all-atom MD simulations of Hsp70-DnaK in different nucleotide-binding states in explicit solvent were performed. We observed the docking of the SBD of DnaK onto the lobe I of its NBD upon ATP-binding: the structure found is an intermediate plausible ATP-bound state, named ATP*, which is compared to several experimental data. A strategy was presented in which the FEL computed from unbiased MD simulations is represented by FEPs of CGDAs of the main chain along the amino-acid sequence of the protein. The FEPs can be quantitatively compared in different nucleotide-binding states by using a similarity index. The analysis of the FEPs allowed us to identify a small network of 27 CGDAs *γ*. The coupling between these 27 CGDAs was deciphered by using dihedral principal component analysis. The conformational change induced by ATP binding was represented by a pathway of minimum energy on the FES built from the two lowest dihedral principal components of the ATP-bound DnaK trajectories. The 27 coordinates correspond to 91 residues which are involved in the interdomain communication upon nucleotide binding to DnaK. Most of these 91 residues revealed by MD were shown experimentally to be relevant for the communication between the NBD and the SBD in the Hsp70 chaperone cycle. The present work also suggests 26 new key residues that could be tested experimentally. For example, the residues A503 to G506, defining *γ_504_*, belonging to the kink between SBD-α and SBD-β, seem to play a key role in the dynamics of the docking as well as for the intrinsic dynamic of the SBD. We found that the conformation of these residues influences the distribution of the distance E430/R547 explaining the structural origin of the variations of this distance observed in EPR upon ATP binding to *E. coli* DnaK [81]. The strategy developed to analyze the long-range effect of ATP binding on the structure and dynamics of *E. coli* DnaK is general and could be applied to decipher allosteric communication in other proteins.

## Methods

### MD simulations

All unbiased all-atom MD simulations in explicit water of *E. coli* DnaK using the NMR-derived structure (PDB ID: 2KHO in which the missing atoms of ARG and TYR were added) [70] were carried out with the GROMACS software package [105] and the GROMOS96 ffG43a1 force field [106], [107]. The time step used in all simulations was 0.001 ps and the list of neighbors was updated every 0.005 ps with the ‘grid’ method and a cut-off radius of 1 nm. The coordinates of all the atoms in the simulation box were saved every 2 ps. The initial velocities were chosen randomly. We used the NPT ensemble with a cubic box. The temperature and pressure were kept to the desired value by using the Berendsen method [108] and an isotropic coupling for the pressure (*T* = 300 K, *τ_T_* = 0.1 ps; P0 = 1 bar, coupling time *τ_P_* = 0.5 ps). The electrostatic term was computed by using the particle mesh Ewald algorithm [109] (with a radius of 1 nm) with the Fast Fourier Transform optimization (on 140 points for DnaK, with an order equal to 4 for the interpolation). The “cut off” algorithm was applied for the non-coulomb potentials with a radius of 1 nm. The system was warmed up for 40 ps and equilibrated for 600 ps with lower restraints, finishing with no restraints at 300 K. We performed 6 MD runs, 2 in each nucleotide-binding state (APO1, APO2, ADP1, ADP2 and ATP1, ATP2), as described in the following subsections. The stability of the protein structure in each MD trajectory was monitored by computing the Root-Mean-Square-Deviation (*RMSD*) between the molecular structure every 2 ps and the initial structure (as done before [23], Fig. S10 A). To reduce the computational cost, the APO and ADP-bound DnaK MD runs were stopped when the *RMSD* was converged to a constant value with a standard deviation lower than 0.5 Å (Fig. S10 B) during at least 50 ns, resulting in six MD runs of different durations. The 2 MD runs of ATP-DnaK were continued after convergence of the *RMSD* up to 0.5 µs in order to explore a possible opening of the SBD which did not occur however on that time-scale.

#### MD runs of APO-Hsp70

The initial structure of the nucleotide-free DnaK (PDB ID: 2KHO) [70] was solvated in a cubic box with 136508 SPC water molecules keeping a minimum distance of 0.9 nm between the solute and each face of the box. We used periodic boundary conditions with an initial value of the box side of 16.112 nm. The charge of the system was neutralized by adding 25 Na^+^ counter-ions. The energy of the system was first optimized with the “steepest descent minimization” algorithm and then by using a “conjugate gradient” algorithm. We performed two runs with different initial conditions. The total simulation time was 151 ns for the MD run APO1 and 360 ns for the MD run APO2.

#### MD runs of ADP-Hsp70

The topology files of the ADP and ATP molecules were constructed with the PRODRG program (version 2.5 BETA) [110]. We added one ADP molecule and one divalent ion (Ca^2+^) to the nucleotide-free DnaK (PDB ID: 2KHO) [70]. The initial coordinates of the ADP molecule were extracted from those of the ADP molecule in the X-ray structure of the isolated NBD of ADP-bound hHsp70 (PDB ID: 1HJO [111]) after alignment of the backbone atoms of the residues 5 to 377 of the hHsp70 NBD on those of the DnaK NBD (PDB ID: 2KHO) by using the VMD software [112]. The protein was solvated with 136488 SPC water molecules [113] in a cubic box of the same dimensions as in the MD runs APO (see above), and 26 Na^+^ counterions were added to neutralize the system. The ADP-bound DnaK initial structures were relaxed during 600 ps with the protein structure restrained to the experimental structure (PDB ID: 2KHO) and with no constraints on the solvent and on the ligands (Dataset S1 in supporting information). We performed two MD runs ADP1 and ADP2 with different initial conditions. The total simulation time was 164 ns for the MD run ADP1 and 151 ns for the MD run ADP2.

#### MD runs of ATP-Hsp70

The ATP-Hsp70 structure was built using the NMR-derived structure of *E. coli* DnaK (PDB ID: 2KHO) [70] with ADP and one divalent ion inserted as described above. The initial coordinates of the ATP molecule was built by adding a phosphate group to the ADP molecule in the free-space available within the NBD of the ADP-bound DnaK structure. The precise initial location of the third phosphate group was not crucial because the ATP-bound DnaK structure was relaxed during 600 ps with the protein structure restrained to the experimental structure (PDB ID: 2KHO) and with no constraints on the solvent and on the ligands (Dataset S2 in supporting information). We compared the ATP-bound construct relaxed after 600 ps with available X-ray structures of isolated NBD of hHsp70 bound to ADP and PO_4_ (PDB IDs: 1S3X [114], 3AY9 [115], 3JXU [116]). The relaxation of the ATP molecule using the present force-field displaced the added phosphate group to a position close to the experimental position of the PO_4_ group in the isolated NBD of hHsp70. Comparison with the new published X-ray full-length ATP-bound *open* structure (PDB ID: 4B9Q) [69], confirmed that the initial relaxed position of the ATP molecule (which mimic the ATP binding position in the full-length *E. coli* Hsp70 in a *closed* state) is realistic. The protein was solvated with 136485 SPC water molecules [113] in a cubic box of the same dimensions as in the MD runs APO and ADP (see above), and 27 Na^+^ counterions were added to neutralize the system. We performed two MD runs ATP1 and ATP2 with different initial conditions. The total simulation time was 553 ns for the MD run ATP1 and 560 ns for the MD run ATP2. The MD runs confirmed that the very precise initial locations of the nucleotides are not so crucial. The X-ray structures provide indeed only a static picture of the binding mode of ADP and ATP. The NBD is dynamics and the nucleotides are fluctuating within the NBD pocket. In the ATP-bound Hsp70 MD simulations, we found that the terminal phosphate groups of ATP and ADP are fluctuating relative to the nucleic base with a *RMSD* varying between 2 Å and 9 Å for ATP and between 2 Å and 6 Å for ADP. The position of the nucleotide fluctuates relative to the NBD (which is itself dynamics) with a *RMSD* varying between 3 Å and 4 Å both for ADP and ATP MD trajectories.

### Definition of the coarse-grained dihedral angles (CGDAs) *γ* and of their free-energy profiles (FEPs)

The conformational fluctuations of the main chain of *E. coli* DnaK were monitored by recording the fluctuations of the CGDAs *γ* [71]. The CGDA *γ* for a residue *i* is the dihedral angle formed by the vectors (virtual bonds) joining 4 successive C^α^ atoms (*i−1, i, i+1 and i+2*) along the amino-acid sequence (Fig. S1 A) [117]. The first dihedral angle along the sequence is *γ_2_* and the last is *γ_N-2_*, where *N* is the total number of residues. Each CGDA *γ_i_* is a local probe of the FEL along the amino-acid sequence of the protein [71].

The FEP of each *γ_i_* is computed from the usual Boltzmann formula:
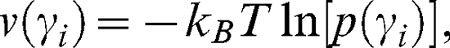
(1)where *k_B_* is the Boltzmann constant, *T* the temperature (300 K) and *p(γ_i_)* is the probability distribution function (PDF) of the CGDA *γ_i_* computed from the MD trajectory (Fig. S1 B) [71]. Note that the experimental NMR structure of *E. coli* DnaK is comprised of 600 residues, starting from residue 4 to residue 603 [70], and is described by 597 angles *γ_i_*, from index *i* = 5 to index *i* = 601.

To quantify the convergence between two FEPs of a same CGDA *γ_i_* in two MD trajectories with the same nucleotide-binding state but with different initial conditions, we computed the similarity index *H* between their associated PDFs [72]:
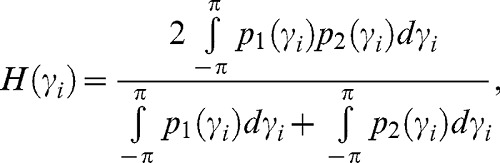
(2)where *p_1_(γ_i_)* and *p_2_(γ_i_)* are two PDFs of the same CGDA *γ_i_* computed from two MD trajectories 1 and 2.

The similarity index *H* varies between 0 (dissimilar) and 1 (identical). Two FEPs, *v_1_(γ_i_)* from the MD trajectory 1 and *v_2_(γ_i_)* from the MD trajectory 2, are considered similar (“converged”) if the index *H* is larger than 0.5.

### Contact map (CM) and contact map difference (CMD)

Distances between all pairs of atoms of the 600 residues were calculated every 2 ps for each MD run (APO1, APO2, ADP1, ADP2, ATP1 and ATP2). The distance matrix between the residues was built by retaining only the smallest atomic distance between the atoms of each residue pair. If this smallest distance was smaller than 4 Å, the residues were said in contact (contact matrix element is 1) and not in contact otherwise (contact matrix element is 0). For each nucleotide-binding state (APO, ADP and ATP), only contacts with at least 50% of occupancy in the two MD runs of *E. coli* DnaK in the same nucleotide-binding state (for example ATP-bound DnaK) and for which the average distance was similar in the two MD runs (for example MD runs ATP1 and ATP2) within 0.5 Å were finally considered, defining the contact map (CM, Fig. S2 A and B) of a nucleotide-binding state (for example ATP). In addition, the difference between the contact maps, the contact map difference (CMD, Fig. S2 C) was computed. For example, the CMD ATP/ADP is the difference between the contact matrices ATP and ADP defined above, and thus reveals the atomic contacts present in the ATP nucleotide-binding state of DnaK, but not in its ADP nucleotide-binding state.

### Dihedral Principal Component Analysis (dPCA)

In dPCA [74], the correlated internal motions of a protein are quantified by a covariance matrix *C_ij_* [Eq. (3)] of the Cartesian components of the two-dimensional vectors ***u*** *(t)* [Eq. (4)] representing the CGDAs:
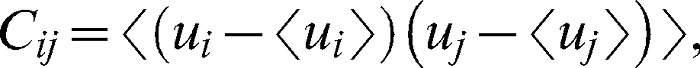
(3)where <…> denotes the average over all sampled conformations and
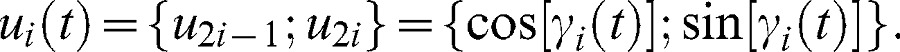
(4)
The diagonalization of the covariance matrix *C_ij_* gives 2*N* ( = number of CGDAs *γ* eigenvalues *λ^k^*, ordered by decreasing value: *λ^1^*>*λ^2^*>…>*λ^2^* and eigenvectors ***e*** *^k = ^{* ***e_1_*** *^k^,* ***e_2_*** *^k^,…,* ***e_27_*** *^k^}^T^*, with ***e_i_*** *^k^ = {e_i_^k^(X);e_i_^k^(*Y*}* where *e_i_^k^(X)* is the Cartesian component *x* of the projection of the eigenvector ***e*** *^k^* on the *i^th^* CGDA (similarly for *e_i_^k^(Y)*).

The contribution of each CGDA *γ_i_* to a mode *k* is the so-called influence [74]:
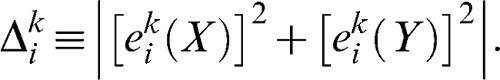
(5)The mean-square fluctuation (*MSF_i_*) of each vector ***u*** *_i_* can be decomposed into collective modes:
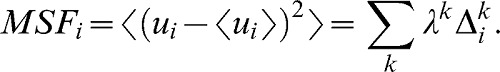
(6)The eigenmodes with the largest eigenvalues *λ^k^* (named slow modes) correspond to the collective modes contributing the most to the *MSF* of the protein [see Eq. (6)]. Therefore, *λ^1^* is the eigenvalue of the collective mode which contributes the most to the structural fluctuations of the CGDAs *γ_i_* in an MD run [72], [74]. Finally, the projection of the MD trajectory on the *k* ^th^ eigenvector **e** ^k^ is named the dihedral principal component *dPC^k^*,
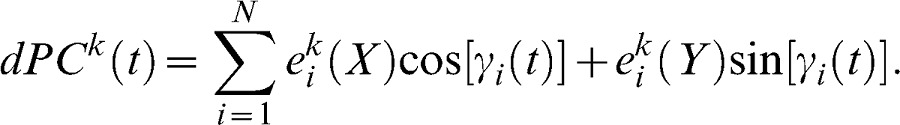
(7)More details can be found in Refs. [72], [74], [118], [119].

### Free-energy surface (FES) from dPCA

The effective two-dimensional FES shown in Fig. 8 was computed from the two-dimensional probability density of *dPC^1^* and *dPC^2^* computed from the covariance matrix of the *subset* of the 27 dihedral angles involved in the conformational change of DnaK (all shown in Fig. 7). The two-dimensional FES computed from the covariance matrix of *all* dihedral angles is indeed irrelevant due to a lack of convergence sampling [94]. Indeed, some dihedral angles are in a diffusive regime. As shown in Refs. [93], [94], the diffusive regime of the internal degrees of freedom of a protein can be measured by the cosine content (CC) of the principal components. A large value (close to 1.0) of the CC means that some of the degrees of freedom are fluctuating as the variables of free random walks on the time-scale of the present MD simulations. The FES built on principal components with CC close to 1.0 shows artifacts, i.e. basins which are signatures of the cosine shape of the principal components and not of an actual minimum of the FES [93], [94], [119]. We computed the cosine content of each principal component [92]. The values of the cosine content computed for *dPC^1^* and *dPC^2^* calculated from the covariance matrix in the space of the 27 dihedral angles having a converged FEP are very small (CC_1_ = 0.11 and CC_2_ = 0.16 for ATP1 trajectory for example). On the contrary, the cosine content of the principal component *dPC^1^* computed in the space of all dihedral angles is very large (CC_1_ = 0.69 for ATP1 trajectory for example).

### Pathway of minimum energy

The Fig. S11 A shows an example of a free-energy surface (FES) generated by projecting a MD trajectory on the essential plane defined by two principal components [Eq. (7)]. In this example, the FES has two minima *A_1_* and *A_2_*. The pathway that connects the two minima *A_1_* and *A_2_* by traveling along the “valleys” of the FES and going over the “passes” (which are saddle-points on the surface and correspond to the transition states) in order to minimize the free-energy difference while moving, is commonly referred to the Pathway of Minimum Energy (PME) [120]. To find the PME for the transition *A_1_→A_2_*, we applied the following methodology. First, an initial guess of the path is made, here the straight interpolation line *d_1_* from *A_1_* to *A_2_*. Secondly, we move from *A_1_* along the direction *d_1_* by 0.1 (which is the bin size defined for the calculation of FES) and draw a segment *p_1_* perpendicular to the direction *d_1_*. Note that the size of the segment *p_1_* is chosen to belong to a circle of diameter *A_1_A_2_*. Finally, we search along the segment *p_1_* the bin with the minimum of free-energy, named *s_1_*. This bin is also considered as the first step of the PME. We repeat the algorithm by constructing a straight interpolation line *d_2_* from *s_1_* to *A_2_*. The algorithm ends after *N* steps when the distance between *s_N_* and *A_2_* is smaller than the size of the bin defined for the calculation of FES. The PME corresponds to the *ensemble {s_j_}_j = 1,..,N_* and to the corresponding free-energy profile *V^PME^(s_j_)* (Fig. S11 B).

## Supporting Information

Dataset S1 Cartesian coordinates of the nucleotide and of the protein for the initial structures of the ADP1 and ADP2 trajectories (Protein Data Bank format is used).
(TXT)

Dataset S2 Cartesian coordinates of the nucleotide and of the protein for the initial structures of the ATP1 and ATP2 trajectories (Protein Data Bank format is used).
(TXT)

Figure S1 **Definition of the coarse-grained dihedral angles (CGDAs)** ***γ*** **.** A: Each angle *γ_i_* is built from the positions of 4 successive C^α^ atoms. The CGDA *γ* varies between −180° and +180° with *γ* = 0° being chosen when C^α^(*i−1*) is *cis* to C^α^(*i+2*), and the clockwise rotation of C^α^(*i+1*)−C^α^(*i+2*) being positive when looking from C^α^(*i*) to C^α^(*i+1*). B: Free-Energy Profile (FEP) of *γ_213_*.
(EPS)

Figure S2 **Contact maps computed from the MD simulations.** A: ATP-bound DnaK. B: ADP-bound DnaK. C: Contact map difference ATP/ADP (See Methods section).
(EPS)

Figure S3 **Dissimilarity index between 1-D FEPs of CGDAs** ***γ*** **.** Histograms showing FEP dissimilarity (*1-H(γ_i_)*) along the amino-acid sequence (from *γ_5_* to *γ_597_*). FEPs of the CGDAs *γ* strongly influenced (*1-H*>0.7), significantly influenced (0.3≤*1-H*<0.7) and weakly influenced (*1-H*≤0.3) by the nucleotide are colored in red, yellow and cyan, respectively. Top panel shows dissimilarities between the FEPs in the APO and the ADP-bound states; middle panel shows dissimilarities between the FEPs in the APO and the ATP-bound states, and the bottom panel shows dissimilarities between the FEPs in the ADP and the ATP-bound states. Subdomain boundaries are indicated with dashed lines.
(EPS)

Figure S4 **Effective FEPs** ***V(γ)*** **of DnaK in** ***k_B_T*** **units in the three different nucleotide-binding states computed from MD simulations.** Only the network of the 27 CGDAs *γ* revealed by the FEP analysis is represented here. The color code is the following: APO, red; ADP, blue and ATP, green.
(EPS)

Figure S5 **Representative structure of each minimum** ***A_1_*** **,** ***A_2_*** **,** ***A_3_*** **,** ***A_4_*** **and** ***A_5_*** **extracted from the FES for the MD run ATP1.** C^α^ atoms belonging to the CGDAs *γ* with large influences are represented by spheres. The color code of the protein structure is the same as in Fig. 1. These figures were prepared with PyMOL [http://www.pymol.org].
(EPS)

Figure S6 **Effective FEPs** ***V(γ)*** **of ATP-bound DnaK in** ***k_B_T*** **units for the network of 27 CGDAs** ***γ*** **.** The values of *V(γ)* for the most probable representative structure extracted from the MD runs ATP1 (*A_3_*) and ATP2 (*B_3_*) are shown with diamonds and triangles, respectively. The FEPs *V(γ)* were translated between −180° and +180° compared with Fig. S4.
(EPS)

Figure S7 **Trajectory along the PME (for the MD run ATP1) of each CGDA** ***γ_i_(s_j_)*** **and their corresponding FEP** ***V(γ_i_)*** **.** Minima and saddle points along the PME (Fig. 9) are represented by gray and red diamonds, respectively. The green diamond corresponds to the minimum *A_3_*.
(EPS)

Figure S8 **Dihedral principal component analysis applied to MD simulations of ATP-bound DnaK.** Influences *Δ_i_^1^* and *Δ_i_^2^* of the 27 CGDAs *γ* extracted from dPCA of the MD run ATP1. The dashed line represents an influence of 10%.
(EPS)

Figure S9 **Representative structures of the minima and of the saddle points observed along the PME on the FES computed from dPCA applied to the MD run ATP1.** At each step *s_j_*, the CGDAs *γ_i_* which have the same conformation as observed in the minimum *A_3_* are represented by green spheres and the others by red spheres. The ATP nucleotide is shown in blue spheres. These figures were prepared with PyMOL [http://www.pymol.org].
(EPS)

Figure S10 **Convergence analysis of MD trajectories.** A: C^α^ *RMSD* with respect to the initial structure computed for the MD runs APO1 (red), ADP1 (blue) and ATP1 (green) up to 250 ns. B: Variance σ of the C^α^ *RMSD* with respect to the initial structure computed for the MD runs APO1 (red), ADP1 (blue) and ATP1 (green) on time windows of 10 ns and up to 250 ns.
(EPS)

Figure S11 **Pathway of minimum energy (PME) for the transition** ***A_1_→A_2_*** **.** A: FES generated for the MD run ATP1 from dPCA. Minima are shown with grey diamonds. The PME is shown with black cross and a red line. Interpolated lines *d* (dashed blue lines) and perpendicular lines *p* (dot-dashed green lines) are shown for the first three steps only. Empty black squares show the intersection between *d* and *p*. B: Free-energy profile *V^PME^* along the PME shown in panel A.
(EPS)

Table S1 **Sequence alignment of human and** ***E. coli*** **Hsp70 s from BLASTp program.** Identities are shown in bold and positives in italic. Residues of *E. coli* Hsp70 relevant for interdomain communication and extracted from the FEL analysis are underlined.
(DOCX)
