# Supplementary material for: Decipher the Mechanisms of Protein Conformational Changes Induced by Nucleotide Binding through Free-Energy Landscape Analysis: ATP Binding to Hsp70
Source: PLoS Comput Biol. 2013 Dec 12;9(12):e1003379. doi: 10.1371/journal.pcbi.1003379 (PMC3861046; doi:10.1371/journal.pcbi.1003379)
Supplement: Table S1 — Sequence alignment of human and E. coli Hsp70 s from BLASTp program. Identities are shown in bold and positives in italic. Residues of E. coli Hsp70 relevant for interdomain communication and extracted from the FEL analysis are underlined. (DOCX) [file pcbi.1003379.s014.docx]

**Table S1.** Sequence alignment of human and *E. coli* Hsp70s from BLASTp program. Residues of *E. coli* Hsp70 relevant for interdomain communication and extracted from the FEL analysis are underlined (91 residues) and positive residues (59 residues) are shown in bold.

| h.  E.C. | 7  5 | IGIDLGTTYSCVGVFQHGKVEIIANDQGNRTTPSYVAFT-DTERLIGDAAKNQVALNPQN  IGIDLG**TT** **S**CV + ++ N +G+RTTPS +A+T D E L+G AK Q NPQ**N**  IGIDLG**TTNS**CVAIMDGTTPRVLENAEGDRTTPSIIAYTQDGETLVGQPAKRQAVTNPQ**N** | 65  64 |
| --- | --- | --- | --- |
| h.  E.C. | 66  65 | TVFDAKRLIGRKFGDPVVQSDMKHWPFQVINDGDKPKVQVSYKGETKAFYPEEISSMVLT  **T+F** KRLIGR**+F** **D** VQ D+ PF++I **D** **V** KG+ A P +IS+ VL  **TLF**AIKRLIGR**RFQD**EEVQRDVSIMPFKIIA-A**DNGDAWVE**VKGQKMA--PPQISAEVLK | 125  121 |
| h.  E.C. | 126  122 | KMKEIAEAYLGYPVTNAVITVPAYFNDSQRQATKDAGVIAGLNVLRIINEPTAAAIAYGL  KMK+ AE YLG PVT AVITVPAYFND+QRQATKDAG IAGL V RIINEPTAAA+AYGL  KMKKTAEDYLGEPVTEAVITVPAYFNDAQRQATKDAGRIAGLEVKRIINEPTAAALAYGL | 185  181 |
| h.  E.C. | 186  182 | DRTGKGERNVLIFDLGGGTFDVSILTID--DG--IFEVKATAGDTHLGGEDFDNRLVNHF D+ G G R + **++DL**GGGTFD+SI+ ID D**G** FEV AT GDTHLGGEDFD+RL+N+  DK-GTGNRTIA**VYDL**GGGTFDISIIEIDEVD**GEKT**FEVLATNGDTHLGGEDFDSRLINYL | 241  240 |
| h.  E.C. | 242  241 | VEEFKRKHKKDISQNKRAVRRLRTACERAKRTLSSSTQASLEIDSLFEGI----DFYTSI VEEFK+ **D+** **+** A++RL+ A E+AK LSS+ Q + + + +  VEEFKKD**QGIDLRND**PLAMQRLKEAAEKAKIELSSAQQTDVNLPYITADATGPKHMNIKV | 297  300 |
| h.  E.C. | 298  301 | TRARFEELCSDLFRSTLEPVEKALRDAKLDKAQIHDLVLVGGSTRIPKVQKLLQDFFNGR TRA+ E L DL ++EP++ AL+DA L + I D+**+LVG**G TR+P VQK + +FF G+  TRAKLESLVEDLVNRSIEPLKVALQDAGLSVSDIDDV**ILVG**GQTRMPMVQKKVAEFF-GK | 357  359 |
| h.  E.C. | 358  360 | DLNKSINPDEAVAYGAAVQAAILMGDKSENVQDLLLLDVAPLSLGLETAGGVMTALIKRN + K +NPDEAVA GAAVQ +L GD **V+D+**LLLDV PLSLG+ET G**GVMT** LI +N  EPRKDVNPDEAVAIGAAVQGGVLTGD----**VKDV**LLLDVTPLSLGIETMG**GVMTT**LIAKN | 417  415 |
| h.  E.C. | 418  416 | STIPTKQTQIFTTYSDNQPGVLIQVYEGERAMTKDNNLLGRFELSGIPPAPRGVPQIEVT +TIPTK +Q+F+T **DNQ** V I V +**GER** **D**N LG+F L G**I** **PAPR**G+PQIEVT  TTIPTKHSQVFSTAE**DNQS**AVTIHVLQ**GERKRAAD**NKSLGQFNLDG**INPAPR**GMPQIEVT | 477  475 |
| h.  E.C. | 478  476 | FDIDANGILNVTATDKSTGKANKITITNDKGRLSKEEIERMVQEAEKYKAEDEVQRERVS FDIDA+GIL+V+A **DK++GK** KITI **G** **L+++**EI++MV+**+AE** **D** E V  FDIDADGILHVSA**KDKNSGKE**QKITIK**ASSG**-**LNED**EIQKMVR**DAEA**N**AEAD**RKFEELVQ | 537  534 |
| h.  E.C. | 538  535 | AKNALESYAFNMKSAVEDEGLKGKISEADKKKVLDKCQEVISWLDANTLAE-KDEFEHKR +N + + + VE+ G K+ DK + + ++ L+ E K E K  TRNQGDHLLHSTRKQVEEAG--DKLPADDKTAI----ESALTALETALKGEDKAAIEAKM | 596  588 |
| h.  E.C. | 597  589 | KELEQVCNPII  +EL QV ++  QELAQVSQKLM | 607  599 |
